# Underdiagnosis of positive resection margins and synchronous peritoneal metastases in locally advanced colon cancer: histopathological reassessment of primary resection in the COLOPEC trial

**DOI:** 10.1007/s00428-025-04065-x

**Published:** 2025-05-16

**Authors:** E.S. Zwanenburg, D. D. Wisselink, C. E. L. Klaver, J. D. W. van der Bilt, J. G. van den Berg, L. L. Kodach, I. D. Nagtegaal, P. J. Tanis, P. Snaebjornsson

**Affiliations:** 1 Department of Surgery, Amsterdam UMC, University of Amsterdam, Meibergdreef 9, Amsterdam, The Netherlands; 2 Cancer Center Amsterdam, Treatment and Quality of Life, Amsterdam, The Netherlands; 3 Department of Surgery, Flevoziekenhuis, Hospitaalweg 1, Almere, The Netherlands; 4 Department of Pathology, Netherlands Cancer Institute, Plesmanlaan 121, Amsterdam, The Netherlands; 5 Department of Pathology, Radboud University Medical Center, Geert Grooteplein Zuid 10, Nijmegen, The Netherlands; 6 Department of Oncological and Gastrointestinal Surgery, Erasmus Medical Center, Dr. Molewaterplein 40, Rotterdam, The Netherlands; 7 Faculty of Medicine, University of Iceland, Reykjavik, Iceland

**Keywords:** Histopathological reassessment, Advanced colon cancer, Underdiagnosis

## Abstract

The aim of this study was to perform histopathological reassessment of primary resections of locally advanced colon cancer (CC) within a randomized controlled trial, with specific focus on surgical margins and synchronous locoregional peritoneal metastases (SL-PM), and to provide learning points for both surgeons and pathologists. All histopathological slides of patients with c/pT4N0-2M0 or perforated CC included in the COLOPEC trial were reassessed and correlated with surgical reports. The COLOPEC trial originally determined the value of prophylactic hyperthermic intraperitoneal chemotherapy (HIPEC). Frequency of positive margins (R +), R + subtypes, and SL-PM and the association with 5-year peritoneal recurrence were analyzed. Histopathological slides of 199 patients were reassessed. R + was present in 28 patients (14.1%), of which 8 occurred at the site of adhesiolysis (originally classified as pT4a in 6). SL-PM was present in 11 cases (5.5%), of which 9 were not recognized or misclassified. Both R + and SL-PM were associated with 5-year peritoneal metastases in cox regression analysis (HR 2.38, 95% CI 1.12–5.04 and HR 5.98, 95% CI 2.69–13.29, respectively). Of 9 patients with peritoneal recurrences detected during re-exploration at 5–8 weeks after primary tumor resection for intended HIPEC, 5 demonstrated either R + and/or SL-PM. This study brings to light previously unnoticed but clinicopathologically relevant aspects of CC pathology retaining to underdetected SL-PM and new R + types. Underrecognition until now probably relates to the complexity of advanced CC specimens, poor communication between surgeons and pathologists, and the low incidence among high volumes of CC specimens. Trial registration: NCT02231086 (Clinicaltrials.gov).

**Supplementary Information:**

The online version contains supplementary material available at 10.1007/s00428-025-04065-x.

## Introduction

Colon cancer resection specimens are among the most common large surgical specimens in pathology laboratories. As such, grossing of these specimens has become a standard procedure, which is probably the reason that gross and microscopic assessment of colon cancer specimens is often considered as being easy and straight-forward. Because the tumor is confined to the colonic wall and mesocolic tissue (including potential regional lymph node metastases) in vast majority of cases, this view might lead to an underestimation of the complexities involved in the assessment of locally advanced colon cancer.

Besides lymph node status, there are two other aspects of primary colon cancer resection specimens that are decisive when dealing with locally advanced cases. These are, firstly, possible tumor involvement of the specimen surface, referring either to the peritoneal surface (pT4a) or the radial surgical margins (from here on referred to as R + when positive), and secondly the presence or absence of synchronous locoregional peritoneal metastases (SL-PM), which are classified as distant metastases (M1) in the TNM system.

Peritoneal metastases (PM) occur when malignant cells extend into the peritoneal cavity in which they seed and form metastases on the peritoneal surfaces. The most common location of PM is in proximity to the primary tumor [1]. PM are often generally referred to as being locoregional, because of spread to either nearby or farther peritoneum, and 4–5% of all colon cancer patients are diagnosed with synchronous PM,^2^meaning that PM are detected at the same time as the primary tumor or within approximately 3 months. About 4–12% of patients with colon cancer are diagnosed with metachronous PM after curative resection [2].

There is no literature on the identification or frequency of unsuspected SL-PM in primary colon cancer resection specimens. The problem of SL-PM being underrecognized and misclassified as other pathology parameters such as for example pT4a or even pT4b (i.e., as being part of the primary tumor), has to our knowledge only been mentioned shortly in a recent review paper on problems in colorectal pathology [3]. It is also important to separate PM from regional lymph node metastases and tumor deposits that develop differently, i.e., via spread inside the mesocolic fatty tissue along neurovascular bundles that anatomically innervate and drain the tumor. This means that tumor lesions in colorectal specimens, separate from the primary tumor, located outside the anatomical draining area and showing peritoneal involvement, are likely PM. As an example, the vascularization/drainage of the greater omentum (via gastroepiploic vessels) is anatomically unrelated to the drainage of colon tumors (via colonic vessels inside the mesocolic fatty tissue) and as such lesions in the greater omentum are PM until proven otherwise. If care is not taken during grossing to separate lesions based on anatomy SL-PM can easily be misclassified within the pN-status, i.e., as regional lymph nodes or tumor deposits. When SL-PM occur on the peritoneal surface of the mesocolon that drains the tumor area, the differentiation between PM and lymph node metastasis/tumor deposit becomes more difficult.

Remarkably, there is very limited attention in daily practice or published literature on histopathological R-status assessment and its prognostic role in colon cancer, unlike rectal cancer. The importance of making distinction between peritonealized (serosal) surface and non-peritonealized surface (a.k.a. the radial surgical margin), has however been emphasized in various guidelines and review papers on both colon and rectal cancer for long [3–5]. The problem probably stems from the fact that it takes training to reliably identify and separate these two surfaces, which may appear similar at gross inspection.

Lack of awareness regarding the R-status and SL-PM among both pathologists and surgeons in colon cancer specimens is likely to result in underrecognition, thereby withholding important information relevant for prognosis and clinical decision-making.

Between 2015 and 2017, the multicenter COLOPEC trial included patients with locally advanced or perforated colon cancer in the Netherlands. For study purposes, all histopathological slides were retrospectively requested for central reassessment as we described previously [6]. The aim of the present study was to determine the frequency and types of R + as well as the frequency of SL-PM in complex colon cancer specimens, to determine discrepancies with the original pathology report, and to evaluate prognostic implications based on 5-year peritoneal metastases rate. Importantly, we also aimed to provide guidance and learning points based on these findings for surgeons and pathologists, to improve their daily practice.

## Methods

### Study population

This study was based on patients with locally advanced colon cancer who were included in the COLOPEC multicenter randomized trial (NCT02231086). The study protocol and results of the primary endpoint of the COLOPEC trial have been published previously [6]. Histological slides of the primary tumor resection specimen of all patients were requested from the tissue archives of 35 laboratories affiliated with the 55 Dutch hospitals in which the resection of the primary tumor was performed, using the infrastructure of the Dutch nationwide pathology databank (PALGA). Data on the original histopathological assessment and staging by the local pathologists was collected from the accompanying pathology reports. The available histopathological slides were scanned (Leica Aperio AT2, × 20 or × 40) and uploaded in a virtual electronic pathology database. Central reassessment was performed by one pathologist (PS) with special interest in advanced colorectal cancer, who was blinded for the original report (except the grossing section) and patients’ individual outcome. For achieving uniformity, a predefined protocol for pathological reassessment was developed. The original surgical reports were requested from the treating hospitals for more detailed information on certain aspects of the surgical procedure with relevance for R-status and SL-PM.

### Definitions of tumor and histopathological characteristics

Resection margin status was defined as positive (R +) when tumor cells extended directly into the non-peritonealized specimen surface in any of the following scenarios: (1) R + , type 1: tumor cells growing into the specimen surface at the site of adhesiolysis (as determined by correlation with the surgical report), represented by a very irregular surface and fresh hemorrhages, often with crushed tissue (mechanical changes), and lacking inflammatory response. (2) R + , type 2: tumor cells growing into (0-μm margin) the mesocolic resection plane, recognized by ink and/or cauterization changes. (3) R + , type 3: tumor cells growing into (0-μm margin) the resection margin of en bloc resected organ(s)/structure(s), recognized by ink and/or cauterization changes. (4) R + , type 4: tumor cells extending into the non-peritonealized outer surface of the specimen at the site of a laceration, tear or tissue defect without any additional tissue changes (“clean specimen injury”) and not related to adhesiolysis, that have occurred either during (e.g., through traction/manipulation) or after the operation (timing usually unknown). A cutoff of 1.0 mm has often been advocated for colon cancer to define R + . This has been done in analogy to rectal cancer, but evidence is lacking for colon cancer. We chose to use true CRM involvement (0 mm) for the definition of R + since the practice of classifying cases with close proximity up to 1.0 mm has the likelihood of diluting the correlation of R-status with recurrence and thus its prognostic impact [7].

SL-PM were considered present based on the combination of information on location and histopathological features. Location should be well separated from the primary tumor, based on either pathology request form (separate container) or gross description. Especially separate nodules in the greater omentum were considered indicative for SL-PM. Regarding histopathological features, lesions with their center above or at the level of the peritoneal surface (using adjacent peritoneum as a reference), typically also with tumor cells on the free peritoneal surface, were considered indicative of SL-PM (see also Supplementary pdf document). Lesions with their center inside the mesocolic fatty tissue, without peritoneal relation, were regarded as conventional (intramesocolic) tumor deposits (TD). Tumor deposits are known to show often relation to vascular and neural structures and this was considered consistent with but not specific of intramesocolic TD. PM may secondarily involve vascular and neural structures, and as such neurovascular involvement is not a discriminating feature.

Tumor type and differentiation were categorized according to the WHO criteria as well/moderately differentiated adenocarcinoma, poorly differentiated adenocarcinoma, mucinous carcinoma, and signet ring cell carcinoma, whereas grading of differentiation was only done in conventional adenocarcinomas, according to the extent of gland formation. For the presence or absence of pT4a, we used an objective cut-off criterion according to Zwanenburg et al. [8]. In short, pT4a was classified as present when tumor cells penetrated the free peritoneal surface or when there was < 0.1 mm distance between tumor cells and the free peritoneal surface. Lymph node status was classified as pN0 (no positive lymph nodes), pN1 (1–3 positive lymph nodes), or pN2 (≥ 4 positive lymph nodes). Tumor deposits were registered separately as absent or present using the 3-mm cut-off of the fifth TNM edition [9] because the fifth edition was still in use in the Netherlands when the COLOPEC trial started.

### Endpoints

The main outcome measures were incidence of R + (with the different subcategories) and SL-PM based on pathological reassessment, and the discrepancy rates with the original report. For prognostic implications of these findings, the rate of 5-year metachronous PM was used as long-term oncological outcome measure. Metastases of the ovaries were also regarded as PM. Peritoneal assessment was performed during follow-up as described in the COLOPEC trial, provided in the published protocol [10].

### Statistical analysis

Descriptive statistics were used to present patient, procedure, and tumor characteristics. Categorical data were presented as numbers with percentages. Five-year metachronous PM rate was determined using Kaplan–Meier analysis, and differences between patient groups were assessed using log rank test. Multivariable analysis was performed using backward stepwise Cox regression. Candidate variables were selected based on current evidence. Significance level was set at a *p*-value of 0.05. All analyses were performed using Statistical Package for Social Sciences (SPSSS) version 26.0 (IBM Corp. Armonk, NY, USA).

### Ethical approval

The COLOPEC trial received ethical approval from the institutional review boards (IRBs) of the Amsterdam University Medical Center. The current sub-study, which utilized data from the COLOPEC trial, was also reviewed and approved by the same ethical committee and by the ethical committee of the Netherlands Cancer Institute (CFMPB433).

## Results

### Patients

From the 202 patients with c/pT4N0-2 colon cancer who were originally included in the COLOPEC trial, 199 could be included for the present study (suppl. Figure 1). Suppl. table 1 presents patient, procedure, and tumor characteristics, as well as histopathological characteristics.

### Pathological reassessment

In total, 28 (14.1%) R + resections were found during central pathological review, compared to five (2.5%) patients with R + resection according to the original pathology reports. Involved margins were located at the site of adhesiolysis (type 1 R +) in 8 patients (28.6%), at the mesocolic resection plane (type 2 R +) in ten patients (35.7%), at the resection margin of an en bloc resected organ (type 3 R +) in six patients (21.4%) and the positive margin was due to an intra/post-operative laceration or tear (type 4 R +) in the remaining four patients (14.3%). In eleven patients (5.5%), SL-PM were found during central review. In two out of the eleven cases, the SL-PMs were correctly diagnosed and reported as such (pM1c). The reason why these patients were included in the COLOPEC trial are because of pre-operative randomization based on cT4 status in one patient, and discussion during multidisciplinary meeting in which the lesion was eventually regarded as cM0 in the other patient. There were no examples of R + or SL-PM overdiagnosis. Take home messages and tips for appropriate clinicopathological assessment of complex colon cancer cases are summarized in Table 1.

**Table 1:** Take home messages and tips for appropriate clinicopathological assessment of complex colon cancer cases

Surgeon: important items that the surgeon should mention on the pathology request form	Report on: • Resection of any en bloc resected structure that is adhesive to the tumor—as this may signify pT4b • Adhesions between the tumor and other structures if these were divided—as this may signify R1 • Intraoperative perforation, laceration, tear, or rupture in the tumor area and if this occurred via the peritoneal or non-peritonealized surface—as this may signify R1 • Suspected synchronous locoregional peritoneal metastases—as this may signify M1
Grossing: tips that aid in detecting SL-PM	Before slicing the specimen: • Inspect the peritoneum for possible locoregional peritoneal metastases. Slice these perpendicular to the peritoneal surface and submit separately • Take notic of lesions that are centered on the peritoneal surface or just under the surface with peritoneal indentations as these may signify peritoneal metastases • Make sure to recognize and separately handle the greater omentum (often only submitted partly) as lesions in this structure can be considered peritoneal metastses ontil otherwise proven (not vice versa)
Grossing: tips that aid in detecting positive resection margins	• Inspect the specimen for more subtle en bloc resected structure that may be adherent to the tumor. Certain adherent structures such as a small part of the greater omentum or abdominal/pelvic wall peritoneum are regularly not reported on the request form • Make a clear distinction between peritonealized and non-peritonealized peritumoral surface. Use ink for the surgically created (non-peritonealized) specimen surfaces and sample these for radial margin assessment (especially if narrow or suspicious margin) • Take notice to the type of perforation/defect (pathophysiological vs. surgical) and their anatomical location (via peritonealized vs. non-peritonealized surface). Note that intraoperative (non-pathophysiological) defects via the peritoneal surface lack signs of peritonitis
Microscopy: tips that aid in recognizing SL-PM	• Take anatomical location and relation to peritoneal surface into account when making a distinction between peritoneal metastases and intramesocolic tumor deposits • An epicenter well within the mesocolic tissue, a relationship with vasculair or neural structures and lack of peritoneal involvement signifies an intramesocolic tumor deposit rather than peritoneal metastasis
Microscopy: tips that aid in recognizing positive surgical margins (R1)	• Cautorized tissue and ink on non-peritonealized surfaces can be used to signify the radial surgical margin of both the mesocolic tissue and en bloc resected structures • Exposed tumor tissue with highly irregular surface, fresh hemorrhages and subtle crushed tissue is likely to signify an area where division of an adhesion between tumor and another structure took place. This signifies R1 (tumor exposed to specimen surface due to technical reasons) and not pT4a which is a pathophysiological process. Contact surgeon for correlation and/or correlate with the surgical report if this is not mentioned on pathology request form

*SL-PM* synchronous locoregional peritoneal metastases

### R + at the site of adhesiolysis

**Fig. 1:**
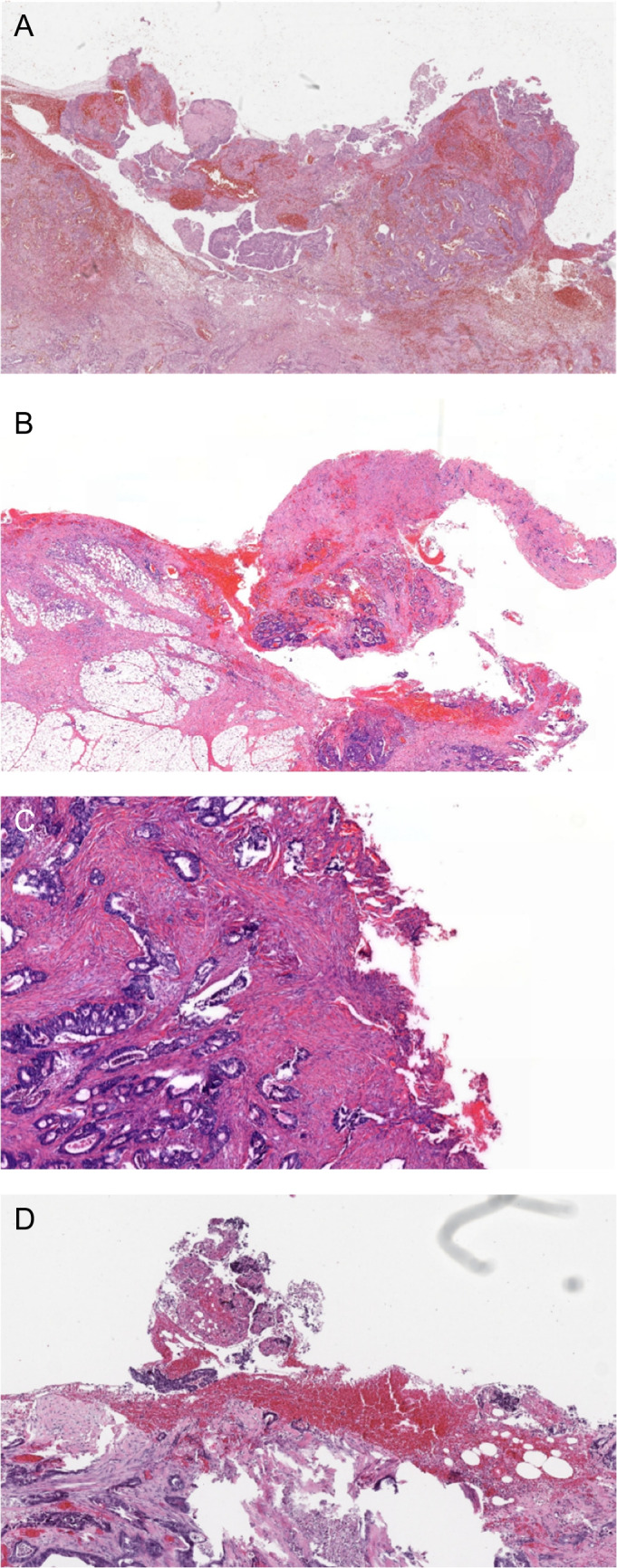
**A**–**D** Representative images of R + at the site of adhesiolysis, H&E stains. **A**, **B** Course tissue fragmentation, lacerations, and fresh hemorrhages, both on the surface as well as in the tissue. **C**, **D** More finely ragged surface, also with fresh hemorrhages

In the eight patients with an R + resection at the site of adhesiolysis on revision, one case was recognized during the initial histopathological assessment, with the remaining cases being classified as R0. In all eight cases, the surgical report contained a description of an adhesive primary colon tumor (seven in the sigmoid colon, one in the cecum) that was detached from the other structure without en bloc resection. None of the pathology request forms included information regarding peritoneal adhesion between the colonic tumor and other structures, and how they were detached. Microscopic examples of R + at the site of adhesiolysis are shown in Fig. 1A–D. These cases were characterized by an irregular specimen surface (course tissue lacerations and/or finely ragged surface), fresh hemorrhages (covering the surface and within the underlying tissue), and crushed tissue in some areas. In six of the eight cases, R + status seems to have been regarded as pT4a. Relevant remarks from the surgical report, pathology request form, and initial grossing are provided in suppl. table 2.

### R + at the mesocolic resection plane

**Fig. 2:**
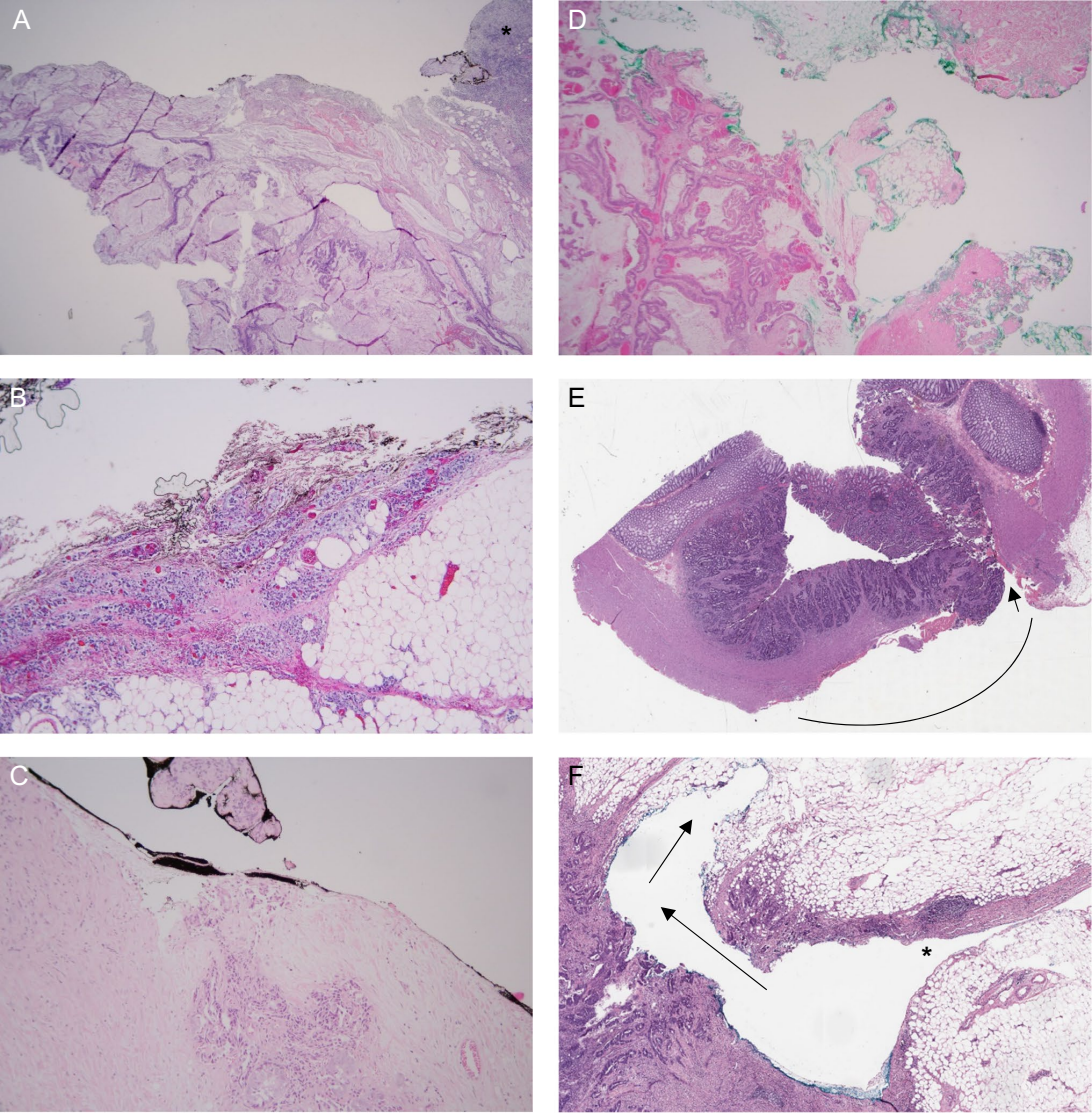
**A**–**F** Representative images of various types of R + resections. **A** Mucinous adenocarcinoma extending extensively into the inked mesocolic resection plane of the specimen, here involved with peritumoral abscess/granulation tissue (asterisk). This area was classified as perforation (without any further clarification), while it would have been more appropriate to classify this area as positive radial resection plane due to extensive transection of the tumor. **B** Signet ring cell carcinoma extending into inked mesocolic resection plane (also recognized as such during initial assessment). **C** Adenocarcinoma extending focally into inked abdominal wall resection plane, not reported on in the original pathology report. **D** Adenocarcinoma extending extensively into inked abdominal wall resection plane, not reported on in the original pathology report. **E** Lacerated specimen at the site of the colonic tumor with large tissue defect (area shown with curved line) and a tear (arrow) extending through the tumor into the bowel lumen. **F** A peritoneal cleft (asterisk) with a tear at the bottom of the cleft, extending into the tumor tissue (arrows). In both **E** and **F**, there is no inflammatory reaction and it is unclear whether these lacerations/tears took place during or after the operation

The ten patients with R + at the mesocolic resection plane had tumors located in the cecum (*n* = 3), sigmoid (*n* = 4), ascending colon (*n* = 1), or appendix (*n* = 2). Two cases were recognized as such during the initial histopathological assessment. Examples are shown in Fig. 2A, B. The slides demonstrated tumor growth into ink on non-peritonealized surface or in cauterized tissue at the mesocolic resection plane. In three cases, there was a peritumoral abscess containing tumor tissue that had been transected; this had been classified as perforation but without noting that this represented intraoperative/iatrogenic specimen perforation at the site of a resection margin.

### R + at the resection margin of en bloc resected structures

In six cases, R + resection at the site of an en bloc resected structure was found. Two were recognized as such during the initial assessment. The tumors were located in the cecum (*n* = 3), sigmoid (*n* = 1), ascending colon (*n* = 1), and descending colon (*n* = 1). The slides demonstrated tumor growth into ink on non-peritonealized surface or in cauterized tissue at the resection plane. Examples of this type of R + are shown in Fig. 2C, D.

### R + due to intra- or postoperative laceration/tear

In four cases, there was a tissue laceration or a tear present, extending into tumor tissue and leaving a “clean” tissue defect, thereby exposing tumor tissue to the outer specimen surface. Tumors were located in the sigmoid (*n* = 3) and ascending colon (*n* = 1). Specimen injury of this type seems to have been regarded as pT4a in three cases, whereas in one case this was reported as iatrogenic perforation. No remarks on intraoperative lacerations or tears were mentioned in the surgical reports. In none of the cases, there was mentioning of adhesions or adhesiolysis. Figure 2E and F both show examples of this type of specimen injury.

### Synchronous locoregional peritoneal metastases

Of eleven patients with SL-PM, 4 had SL-PM located in the greater omentum. In three of these cases, the omentum was submitted separately from the colectomy specimen (two within the same container, one in a separate container), and in the fourth case, the omentum was part of the main specimen. In one case, the SL-PM was located on the appendix, in one case on the distal ileum, and in the remaining five cases on the peritoneum covering the mesocolic tissue of the colectomy specimen. Out of the eleven cases with SL-PM, there were eight cases that demonstrated a single SL-PM while a total number of three, six, and eight SL-PM were found in the other three cases, respectively.

**Fig. 3:**
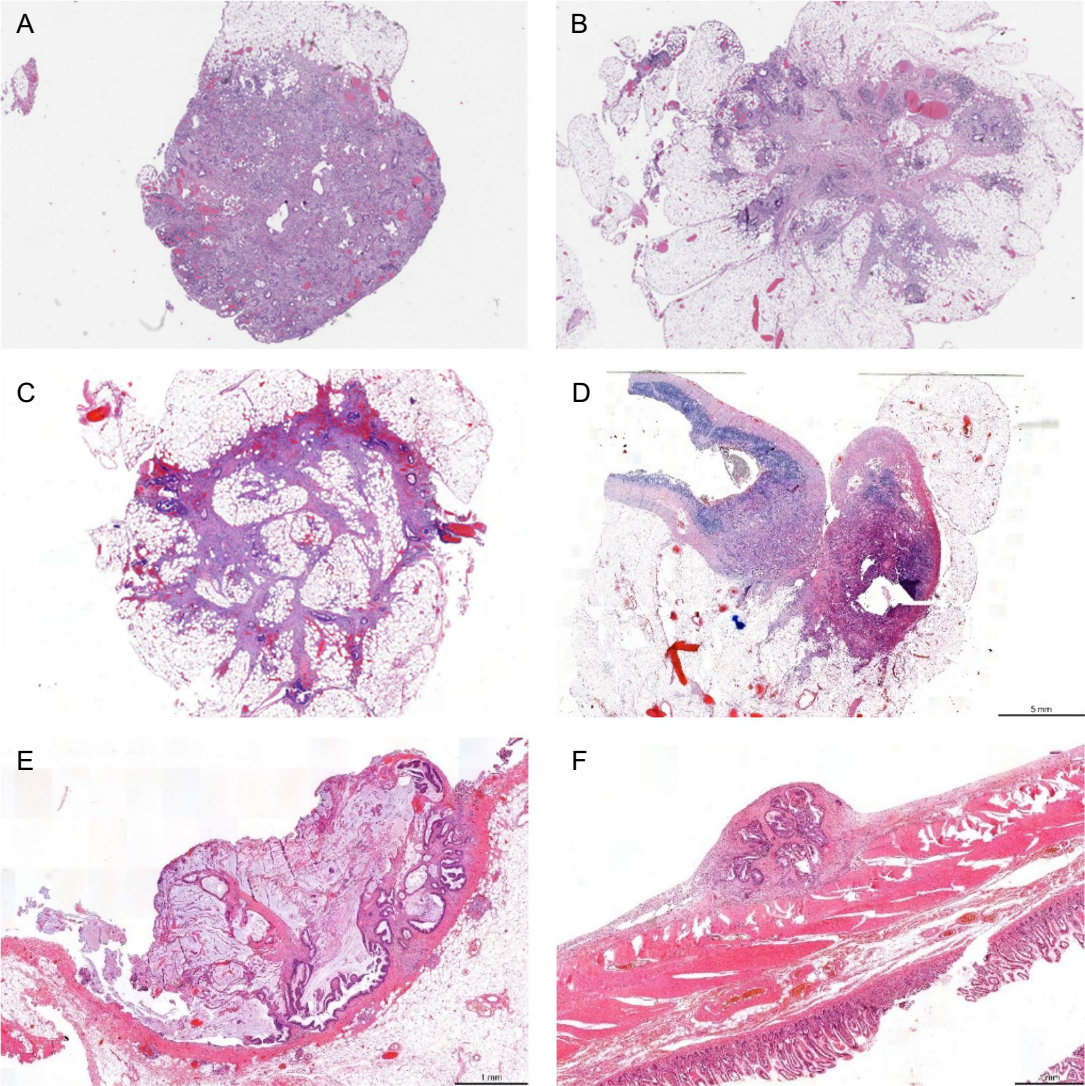
**A**–**F** Representative images of synchronous locoregional peritoneal metastases (SL-PM), H&E stains. A, B, and C were SL-PM located in the greater omentum. The omental tissue is characterized by and recognizable by small fatty lobules, each covered with peritoneum (**B** and **C**). **D** is a SL-PM growing into the appendix close to the appendiceal top. **E** and **F** are from cases with SL-PM located on the mesocolic fatty tissue and the distal ileum, respectively. These two PM are recognizable by an epicenter located at or largely above adjacent peritoneal surface. The SL-PM in **E** was marked with a suture by the surgeon but only vaguely mentioned in the pathology report as a lesion without any specification/labeling. Peritoneal metastases that have an epicenter *below* the peritoneal surface, such as case **D**, are more generally more difficult to recognize

Regarding the interpretation and reporting of the SL-PM in the original pathology reports, there was no discrepancy with the original report in two patients as already mentioned. In five out of eleven cases, the SL-PM were submitted during grossing as lymph nodes and/or microscopically classified as tumor-positive lymph nodes or (intramesocolic) TDs. In one case, the SL-PM was originally regarded as synchronous primary adenocarcinoma of the appendix, but based on central review, this appeared to be a peritoneal metastasis from the colon. Since this lesion had been considered a second primary tumor, we added in this case molecular clonality analysis, which verified the central pathology assessment. In the remaining three cases, the SL-PM was missed, disregarded, or only vaguely mentioned without any labeling. Correlation with the surgical reports revealed that in two of eleven patients with SL-PM, it was mentioned in the operative report that peritoneal abnormalities were observed, but this intraoperative finding was not conveyed to the pathologist. Figures 3A–F show examples of unrecognized SL-PM. Detailed information on each case is provided in suppl. table 3.

### Metachronous peritoneal metastases

Suppl. Figure 2A shows the Kaplan–Meier curves for metachronous PM in patients with R0 versus R + resections based on central review; 5-year PM rate was 23.3% and 33.6% (*p* = 0.179), respectively. The metachronous PM curves of patients with different R + types are shown in suppl. Figure 2B; 5-year PM rates were 50.0% for R + type 1, 33.5% for R + type 2, 16.7% for R + type 3, and 25.0% for R + type 4. Suppl. Figure 3 shows 5-year metachronous PM rates of patients with SL-PM. Both the presence of R + (HR 2.38, 95% CI 1.12–5.04) and SL-PM (HR 5.98, 95% CI 2.69–13.29) were independently associated with 5-year metachronous PM in multivariable analysis (suppl. table 4).

### Early peritoneal metastases in the experimental arm

As previously published in the paper on the primary outcome of the COLOPEC trial, nine patients in the experimental arm had peritoneal metastasis diagnosed at the time of abdominal re-exploration, 5–8 weeks after the index surgery, in which it was decided not to continue with the intended prophylactic HIPEC treatment.^4^ Suppl. table 5 presents characteristics of those patients. Of nine (17.9%) patients with early recurrence, four had an R + resection, and two patients had SL-PM present (in total five patients since one patient had both). One patient had signet cell carcinoma histology, seven had pT4a, and in six cases, pN2 was demonstrated. No remarks on SL-PM were found in the original surgical reports in all nine patients.

## Discussion

Central pathological review of the primary tumor of 199 patients with locally advanced colon cancer who were included in the COLOPEC trial revealed high discrepancy rates with the original pathology report. Positive radial surgical margins (R +) were found in 28 cases, and this was originally reported in only five of them. Furthermore, we have observed that SL-PM are easily missed during histopathological assessment of locally advanced colon cancer, given the fact that only two of eleven such cases were initially diagnosed.

Positive resection margins are apparently difficult to recognize and are often interpreted as pT4a. This is probably partly related to a lack of awareness, both during grossing and microscopic assessment [3]. Also, we have seen in our practice that the distinction between peritonealized (serosal) surface and non-peritonealized specimen surface (i.e., the radial resection margin) takes training and proper anatomical knowledge. Untrained pathology personnel may easily confuse these two. In a supplementary pdf document, we show examples of how these two specimen surfaces can be identified from each other. This is important because, R + and pT4a have distinctly different implications, especially regarding surgical quality assurance. R + indicates an incomplete oncological resection and is a strong predictor of worse oncological outcomes [11]. In this cohort, we found R + resections to be independently associated with 5-year metachronous peritoneal metastases (HR 2.38, 95% CI 1.12–5.04). Moreover, for surgeons to be able to improve their surgical technique, it is vital to get correct feedback on the surgical margin. In particular, we noticed that 8 (4.0%) patients had R + at the site of adhesiolysis where surgeons seem to have performed blunt dissection or cut through adhesions containing tumor tissue.

As a general important oncological principle, adhesions to a colonic segment that contains a primary cancer should always be approached with the intention to perform an *en bloc* resection with adjacent organs/structures, as true tumor invasion in the presence of such adhesions is confirmed by the pathologist in 34 to 63% of cases [12–15]. It is impossible for the surgeon to distinguish if adhesions to surrounding tissues are due to inflammation or contain cancer. Furthermore, the surgeon should inform the pathologist via the pathology request form about issues related to the surgical margins, especially if adhesiolysis at the site of tumor has been performed. Without such information, pathologists are likely to confuse R + at the site of adhesiolysis with pT4a. We show in the current study that certain histopathological changes may alert the pathologist that adhesiolysis could have been performed. These are signs of tissue injury and include irregular specimen surface in combination with fresh hemorrhages and crushed tissue. If these features are encountered the pathologist may also check the surgical report (as we did in the current study) or contact the surgeon for further information and verification.

In ten patients (5%), the mesocolic plane margin was positive. This has previously especially been described in tumors of the cecum and proximal ascending colon in 7 to 8% [16, 17] although the present study shows that this may also occur in other colonic segments. In the FOXTROT trial, the R1 rate was significantly reduced by induction chemotherapy. This seems a valid treatment strategy in locally advanced colon cancer to reduce positive margins by preoperative downstaging [18].

In the current study, we found that so-called *perforations* pose a classification problem for pathologists regarding pT4a and resection margins. The reason is that the term perforation is used for various types of physiological perforations as well as mechanical or technical changes to the surgical specimen, occurring during or after surgery. As such, we found that the following scenarios were referred to as perforation and subsequently regarded as pT4a. These are, firstly, tumor rupture with risk of spillage occurring clearly during surgery. Secondly, positive mesocolic resection planes extending through tumor tissue, typically when involving peritumoral abscess, are also sometimes referred to as perforation. Thirdly, perforation is also used to denote cases demonstrating “clean specimen injuries” created by tear or laceration. We argue that pathologists should take care to differentiate between (A) pT4a where tumor cells penetrate microscopically to the peritoneal surface (physiological process) and (B) exposure of tumor tissue to the specimen surface for technical (non-physiological) reasons, which should in most cases be classified as R + . With that being said, it is unfortunate that the term perforation has been used in the TNM literature to describe both microscopic penetration of tumor cells through the peritoneum (pT4a) as well as for free, gross perforation of colorectal carcinoma into the peritoneal cavity. Based on the current study, we advocate that the term perforation should be more strictly defined within the TNM literature, and it should not be used to describe microscopic peritoneal penetration of tumor cells. Also, in order to avoid confusion with pT4a, we propose that the various types of R + are clearly specified within the TNM literature as well.

In this study, we found that SL-PM are commonly confused with (intramesocolic) TDs, which might have also been classified as lymph nodes in the past. This misclassification has important negative consequences since TDs belong to the pN category of the TNM system, similar to regional lymph node metastases, while PM signify distant metastases (pM), leading to very different prognosis and treatment strategies. In this cohort, we found the presence of SL-PM to be independently associated with 5-year metachronous peritoneal metastases (HR 5.98, 95% CI 2.69—13.29). Sometimes peritoneal lesions were recognized during grossing but were subsequently overseen as such during microscopic analysis and misclassified as lymph node metastases (or tumor deposits). Lesions within the greater omentum, however, are usually peritoneal metastases and can be regarded as such until proven otherwise. Recognizing the greater omentum as a separate anatomical structure is thus of importance during grossing for staging purposes. Also, the surgeon should always report on the (possible) presence of peritoneal lesions within the resected specimen to the pathologist and mark these with a suture or submit in a separate container where feasible. We noticed that sometimes it was mentioned in the surgical reports that there were peritoneal lesions present, but this was not communicated to the pathologist on the pathology request form. Consequently, these lesions were not recognized as being peritoneal metastases, resulting in understaging (M0 instead of M1). These cases demonstrate serious consequences of poor communication between the operating room and pathology. We believe that there is clear need for addressing this problem in quality assurance programs for colorectal cancer surgery. Structured pathology request forms may help.

Limitations of the current study are that we have introduced a new way of classifiying R + . Especially, the current paper contains the first description of R + at the site of adhesiolysis. As such, some aspects of our classification might be controversial and will need more research before wider acceptance is achieved. As such, the new R + types were unlikely to be reported in the original pathology assessment. The 4 types of R + , as we propose in the current paper, especially type 1 and 4, need close correlation with surgical reports and preferably discussion with the surgeon—and this may not be easily achievable in some cases or settings.

In conclusion, this central revision of histopathology in the COLOPEC trial revealed that R + , especially variants 1 and 4 as described for the first time in this paper, and SL-PM go easily unnoticed and may be misclassified. We have shown that both R + and SL-PM are adverse prognostic parameters that are associated with peritoneal recurrence. The findings indicate that quality can be improved by better adherence to the oncological principles of en bloc resection and better communication with the pathology departments regarding potential issues regarding surgical margins and intraoperative detection of peritoneal abnormalities. In addition, we point out morphological features that will facilitate appropriate classification of tumor in relation to surgical margins, peritoneal surface, and tumor surface.

## Supplementary Information

Below is the link to the electronic supplementary material.

Supplementary file1 (PDF 2.75 mb)

Supplementary file2 (XLSX 18.1 kb)

Supplementary file3 (PDF 682 kb)
