# Supplementary material for: Underdiagnosis of positive resection margins and synchronous peritoneal metastases in locally advanced colon cancer: histopathological reassessment of primary resection in the COLOPEC trial
Source: Virchows Arch. 2025 May 16;487(4):787–97. doi: 10.1007/s00428-025-04065-x (PMC12546376; doi:10.1007/s00428-025-04065-x)
Supplement: Supplementary file 1 — (PDF 2.75 mb) [file 428_2025_4065_MOESM1_ESM.pdf]

Figures to aid the recognition serosa/peritoneum versus radial resection margin in CRC resection specimens

Supplementary data to the paper:

Under-diagnosis of positive resection margins and synchronous peritoneal metastases in locally advanced colon cancer: histopathological reassessment of primary resection in the COLOPEC trial.

ES Zwanenburg MD1,2, DD Wisselink MD1,2, CEL Klaver MD, PhD1,2, JDW van der Bilt MD, PhD1,2,3, JG van den Berg MD, PhD4, LL Kodach MD, PhD4, ID Nagtegaal MD, PhD5, PJ Tanis MD, PhD1,2,6, P Snaebjornsson MD, PhD4,7, on behalf of the COLOPEC collaborators^

Ileocecal resection

- The cecum has anatomic variation in which it varies to which extent it is covered by peritoneum dorsally.
- In figures on slide 3 the cecum and a part of the proximal ascending colon is covered with peritoneum dorsally.
  - Where the peritoneal covering stops dorsally and the mesocolic (“radial”) resection margin takes over is marked with white arrows.
- In slide 4 the figures (from another specimen) show this transition at a much lower level (which is more common), also marked with white arrows.

Ileocecal resection

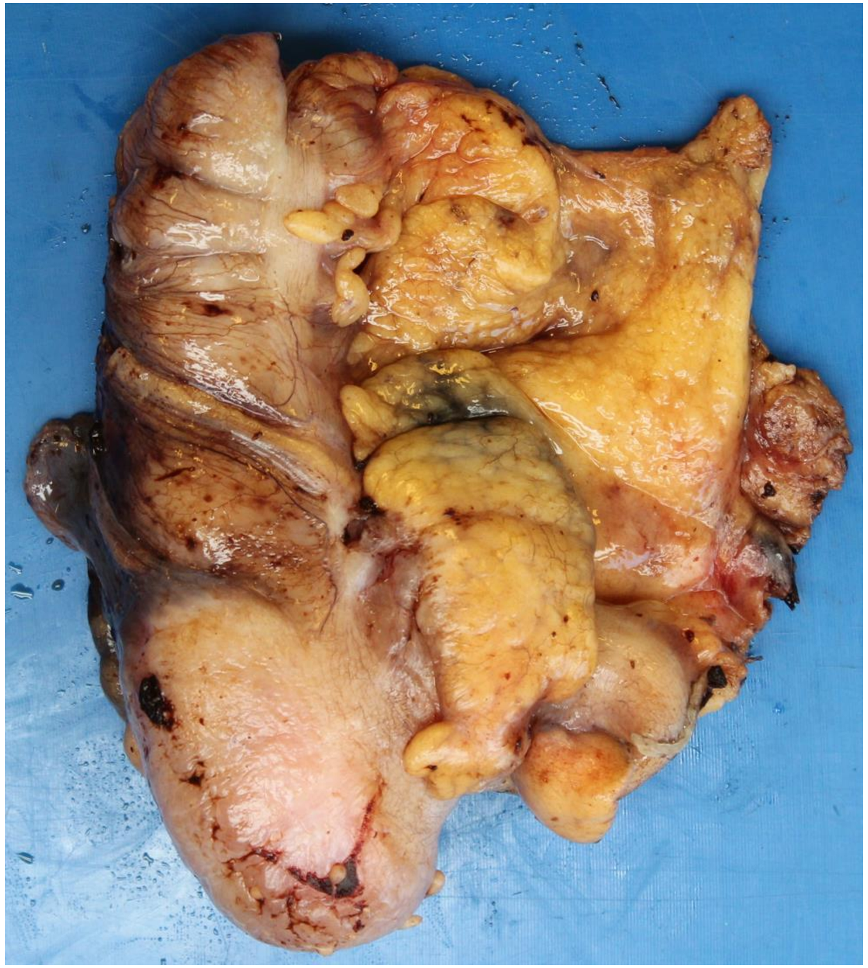

Ventrally there is peritoneal covering

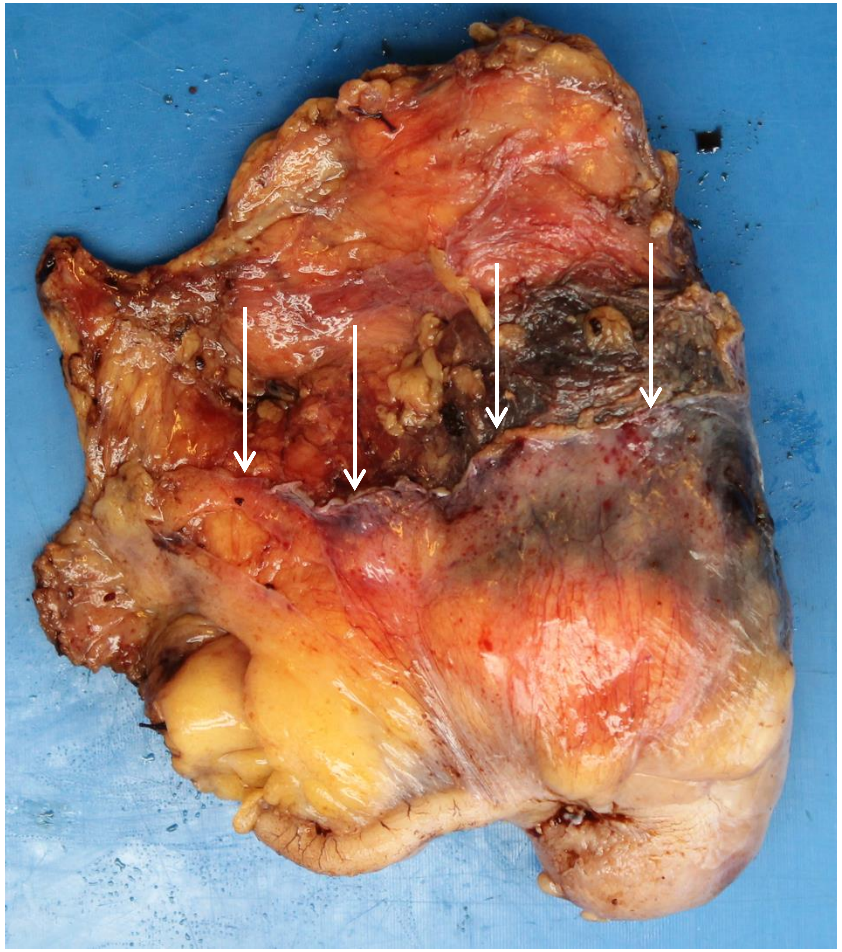

Dorsally there is in this case partial peritoneal covering

Ileocecal resection/right hemicolectomy

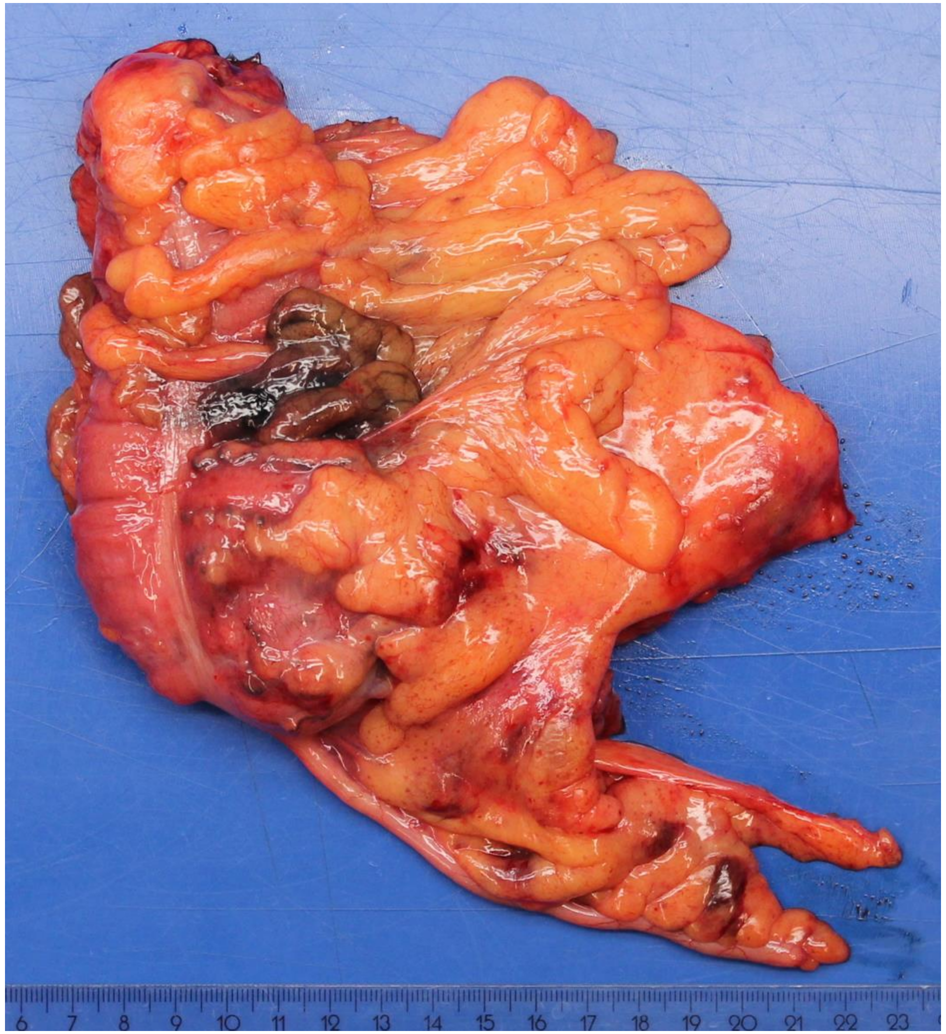

Ventrally there is peritoneal covering

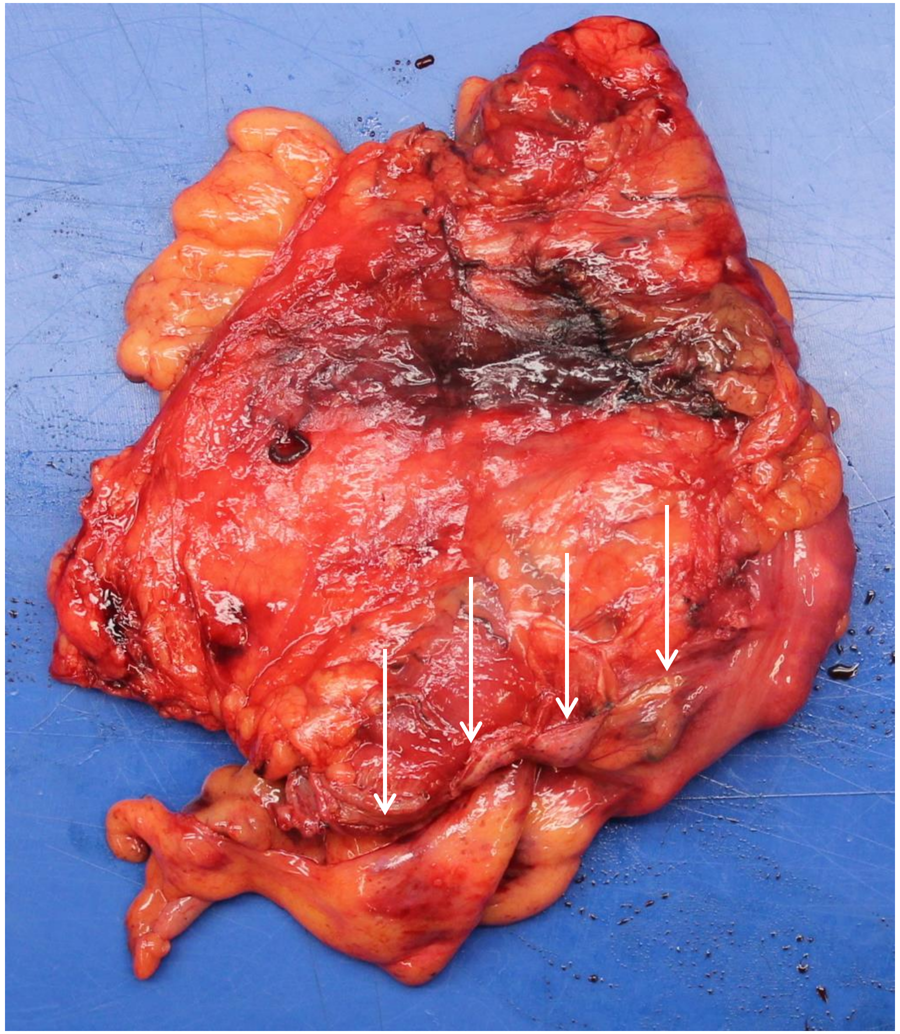

The dorsal surface is mainly radial/mesocolic resection margin

Right hemicolectomy

- The figures in slide 6 show a right hemicolectomy specimen
  - As in figure 4 the cecum does not have a peritoneal covering dorsally
- The radial/mesocolic resection margin is in this case at the level of the mesocolic fascia, which is well visible in this specimen (see dorsal view).
- We inked the radial/mesocolic resection margin at the level of the tumor and then the tumor area was sectioned (see slide 7)
  - In the sections it can be appreciated that the tumor grows at most superficially into the mesocolic fatty tissue and that the inked radial/mesocolic resection plane is far off.

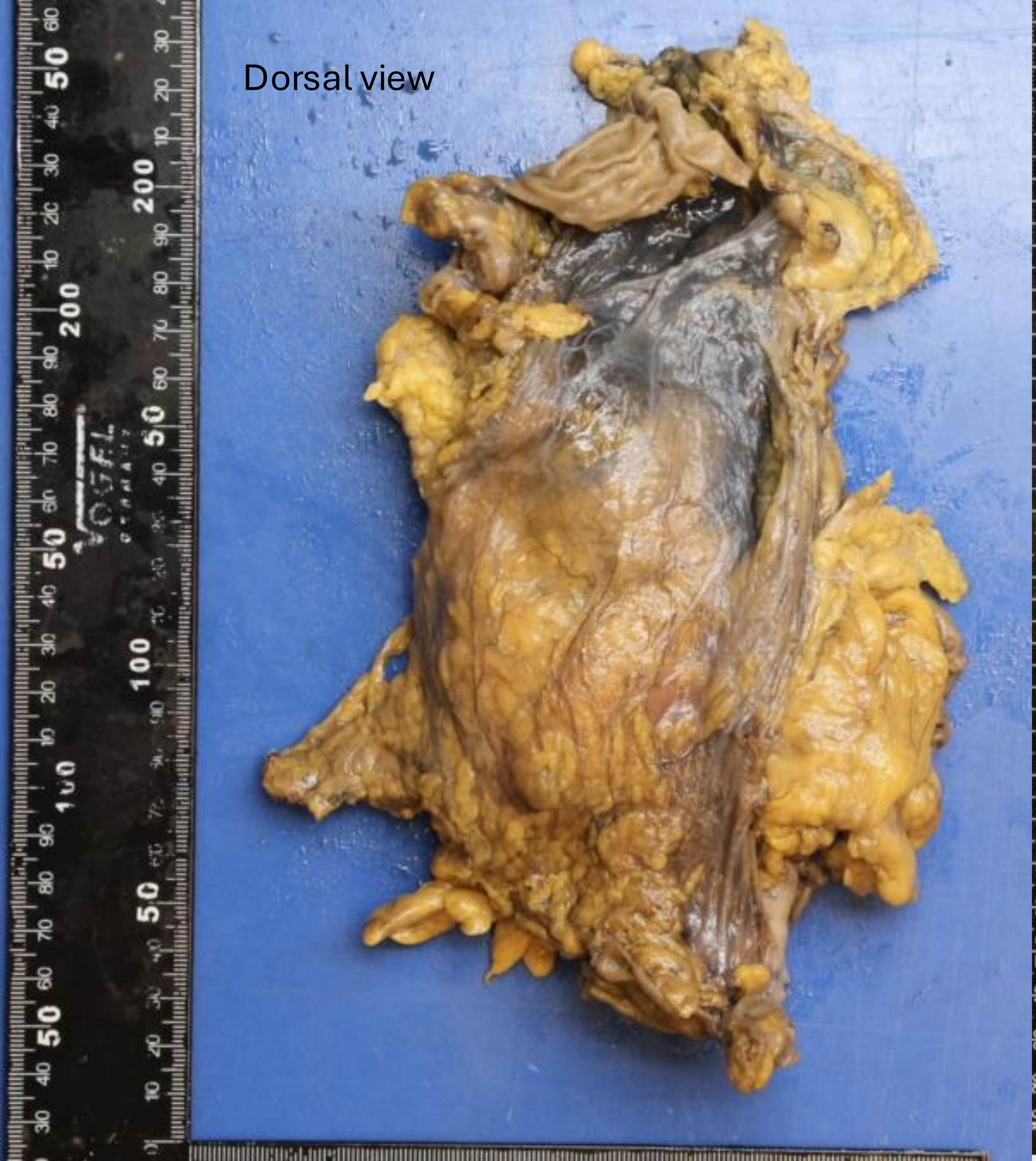
Dorsal view

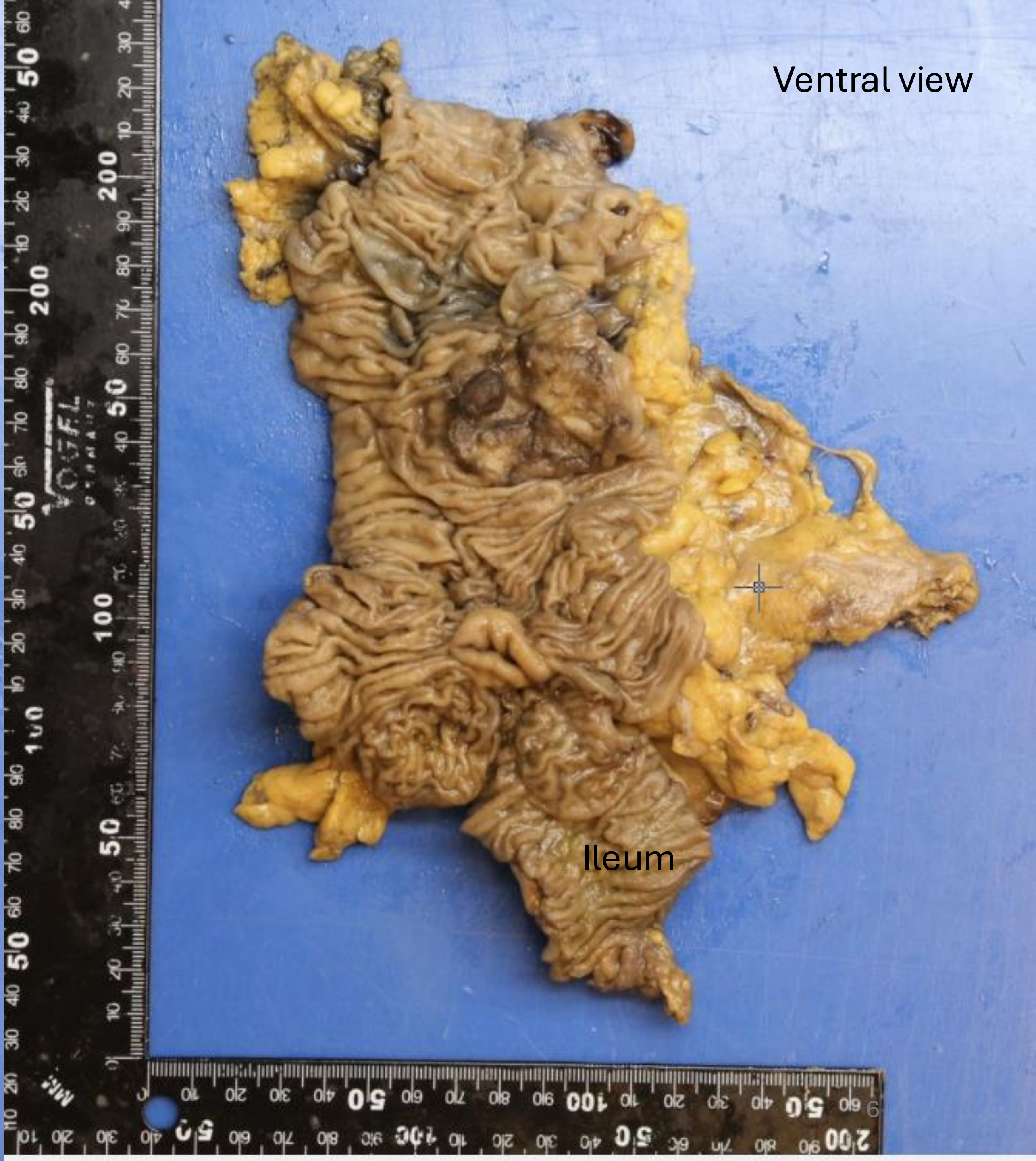
Ventral view
Ileum

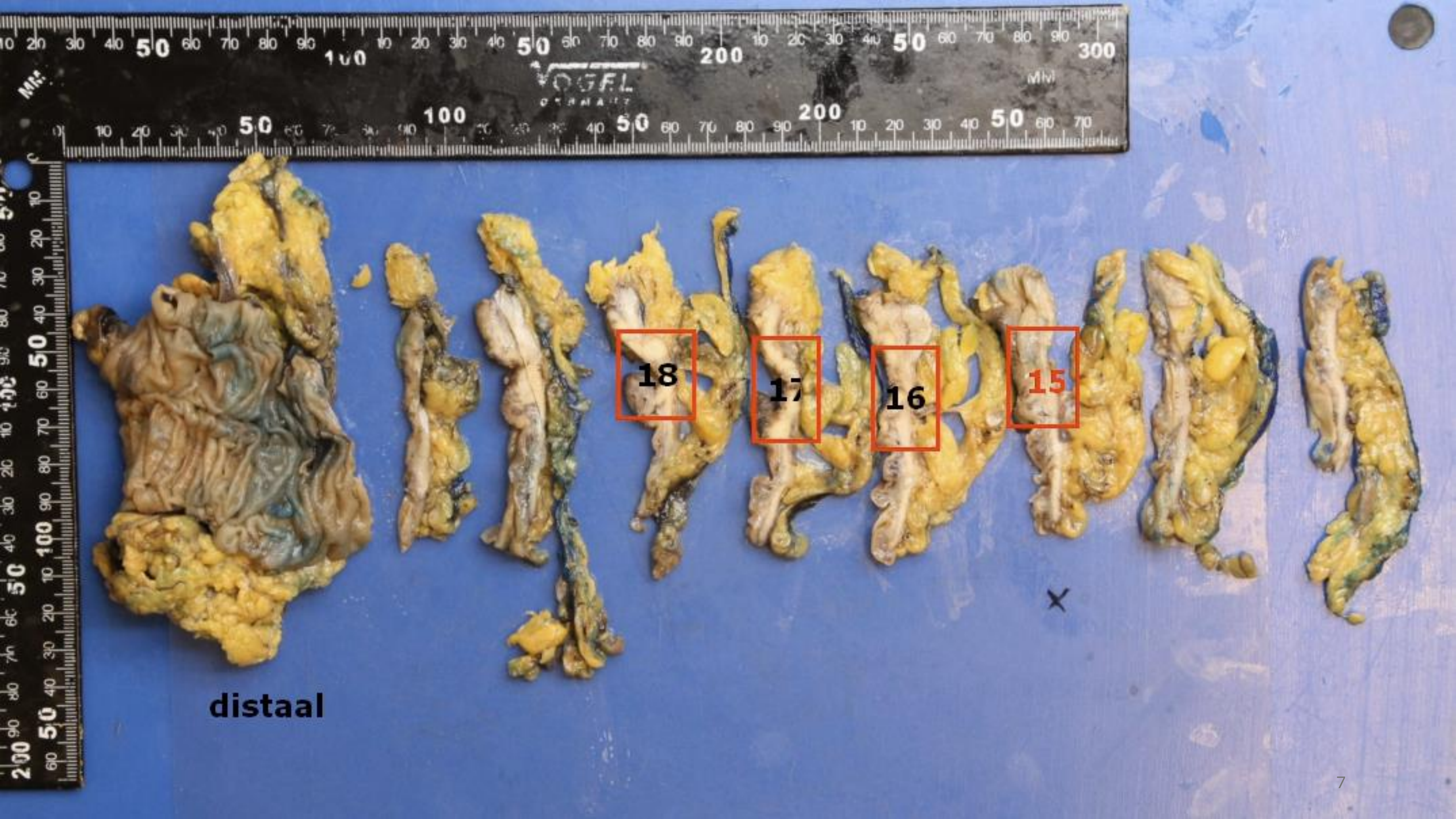
18
17
16
15
distaal

Left hemicolectomy

- The figures in slide 9-12 show a left hemicolectomy specimen en-bloc with a piece of peritoneum from the adjacent lateral bowel wall. This small piece of peritoneum was stuck to the colonic tumor via peritoneal adhesion.

- The figures show how the different resection planes of different structures can be recognized and inked separately

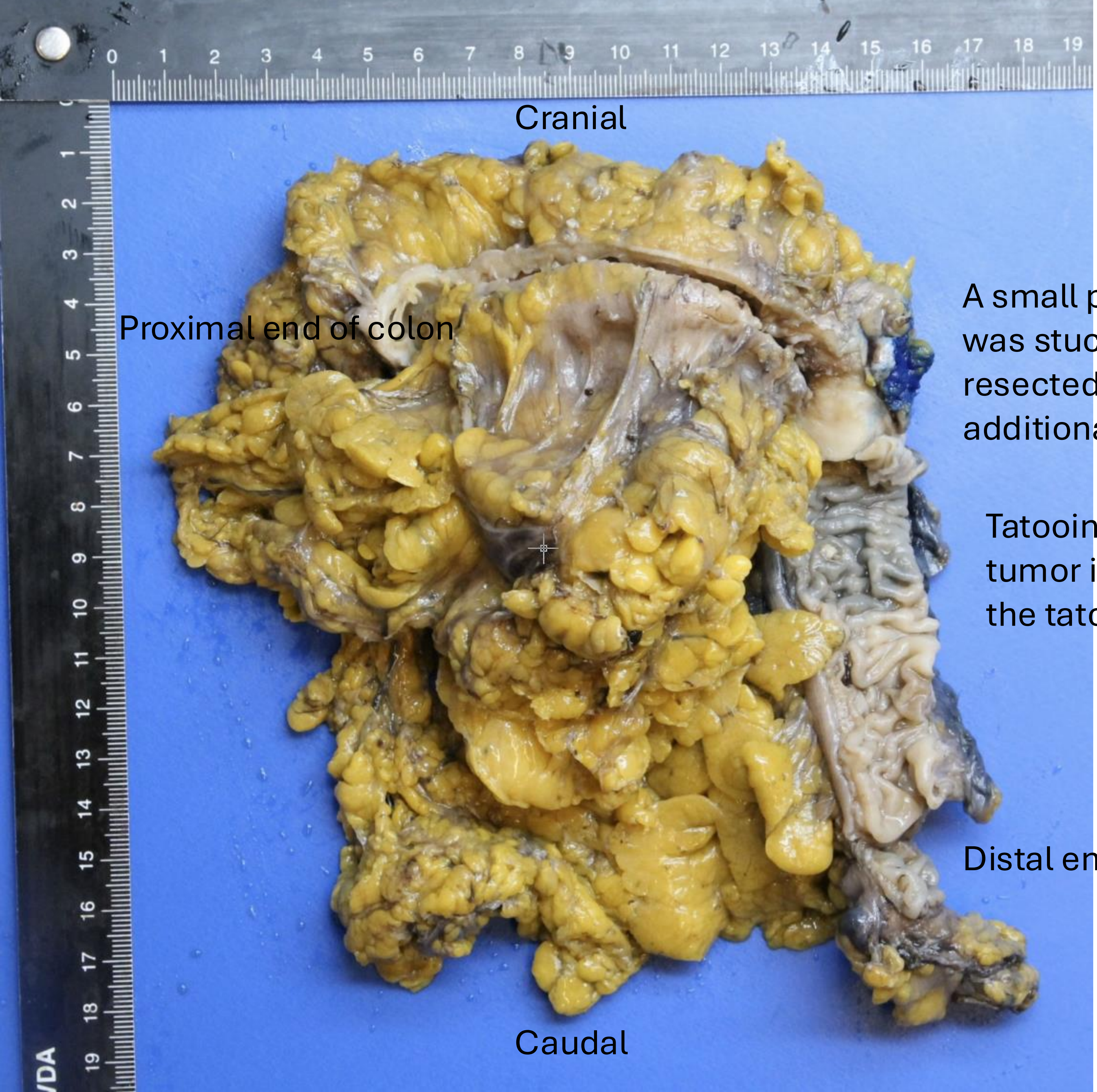

Ventral view

A small piece of lateral left abdominal wall peritoneum was stuck to the colonic tumor via adhesion and resected en-bloc. We inked the resection margin of this additional specimen blue.

Tatooing of the mucosa distally of the tumor. The tumor is poorly visible but it is located just proximal to the tatoo

Slightly distally from the tumor (where the bowel is tattooed black) you can appreciate where the peritoneum stops (anatomically in the left lateral paracolic gutter) and the radial/mesocolic resection margin takes over

Side view

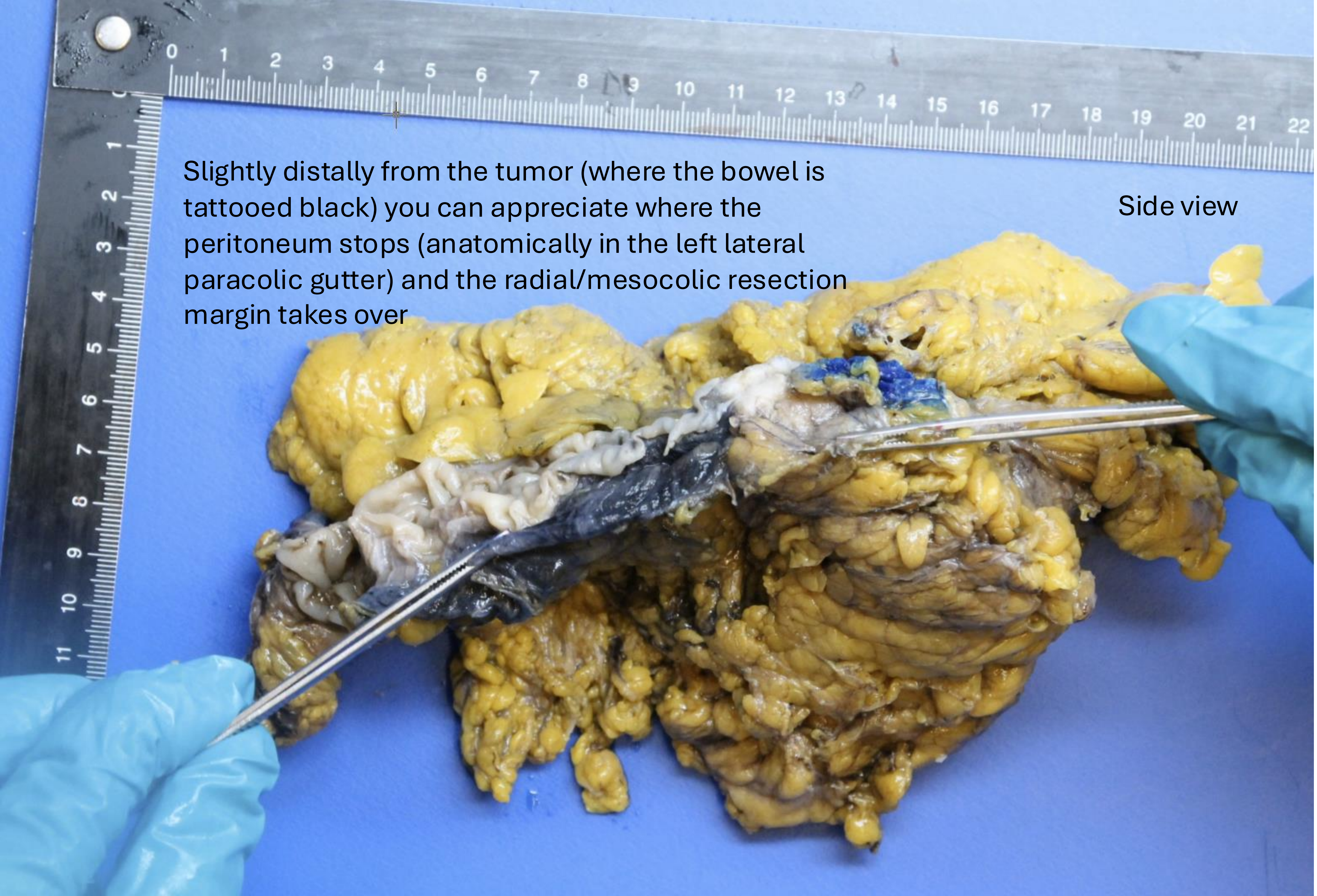

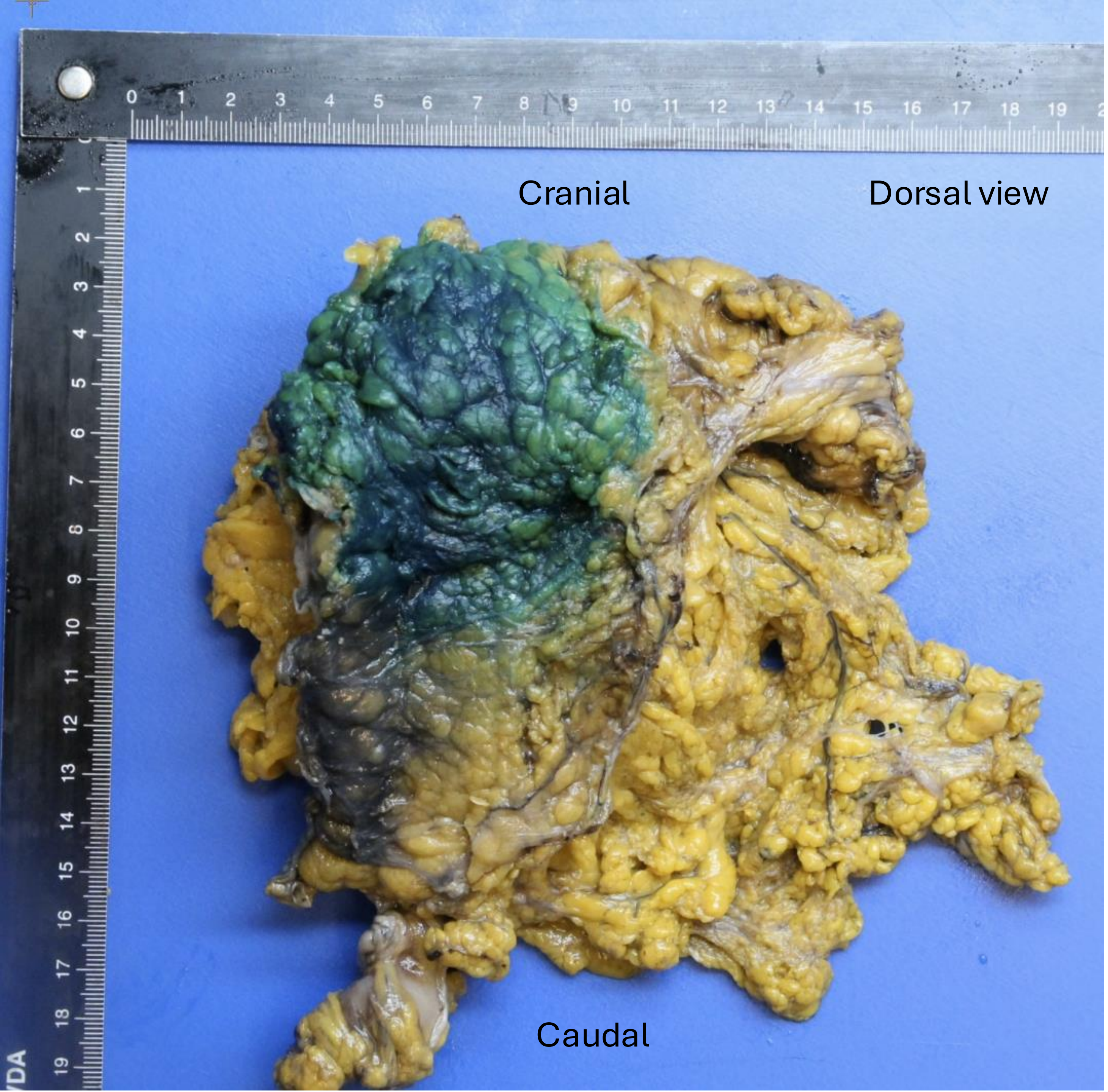

Dorsal view

Here we inked the radial/mesocolic resection margin green at the level of the tumor

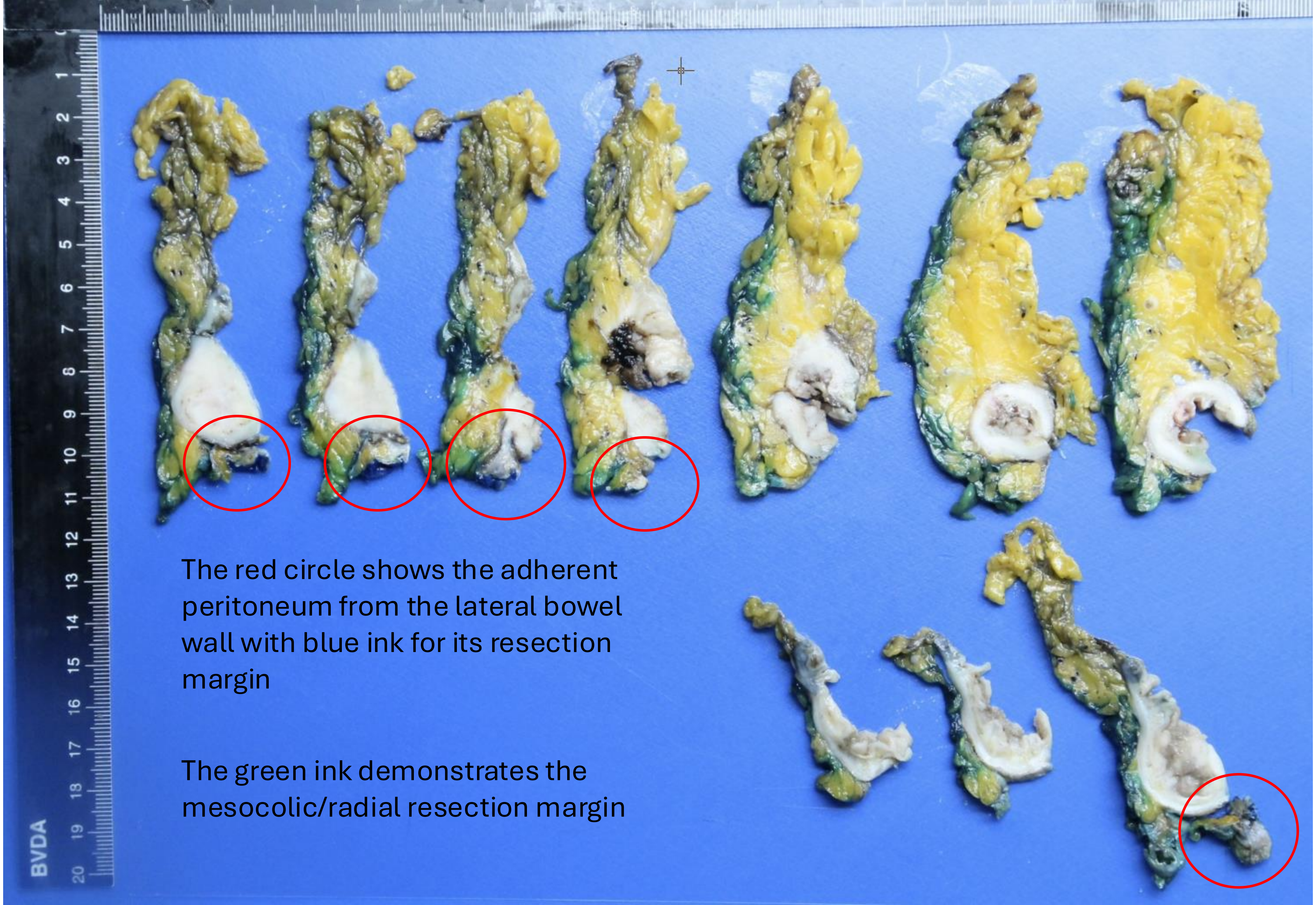

The red circle shows the adherent peritoneum from the lateral bowel wall with blue ink for its resection margin

The green ink demonstrates the mesocolic/radial resection margin

Sigmoid resection

- The figure in slide 9 shows sigmoid resection up-side-down with the taenia libera facing the table the mesocolic (“radial”) resection margin of the sigmoid mesocolon facing the camera
- The arrows point to where the peritoneal covering stop on each side, with inbetween the resection margin of the sigmoid mesocolon
- The mesocolon of the central part of the transverse colon has the same anatomic configuration (see slide 10).

Sigmoid resection

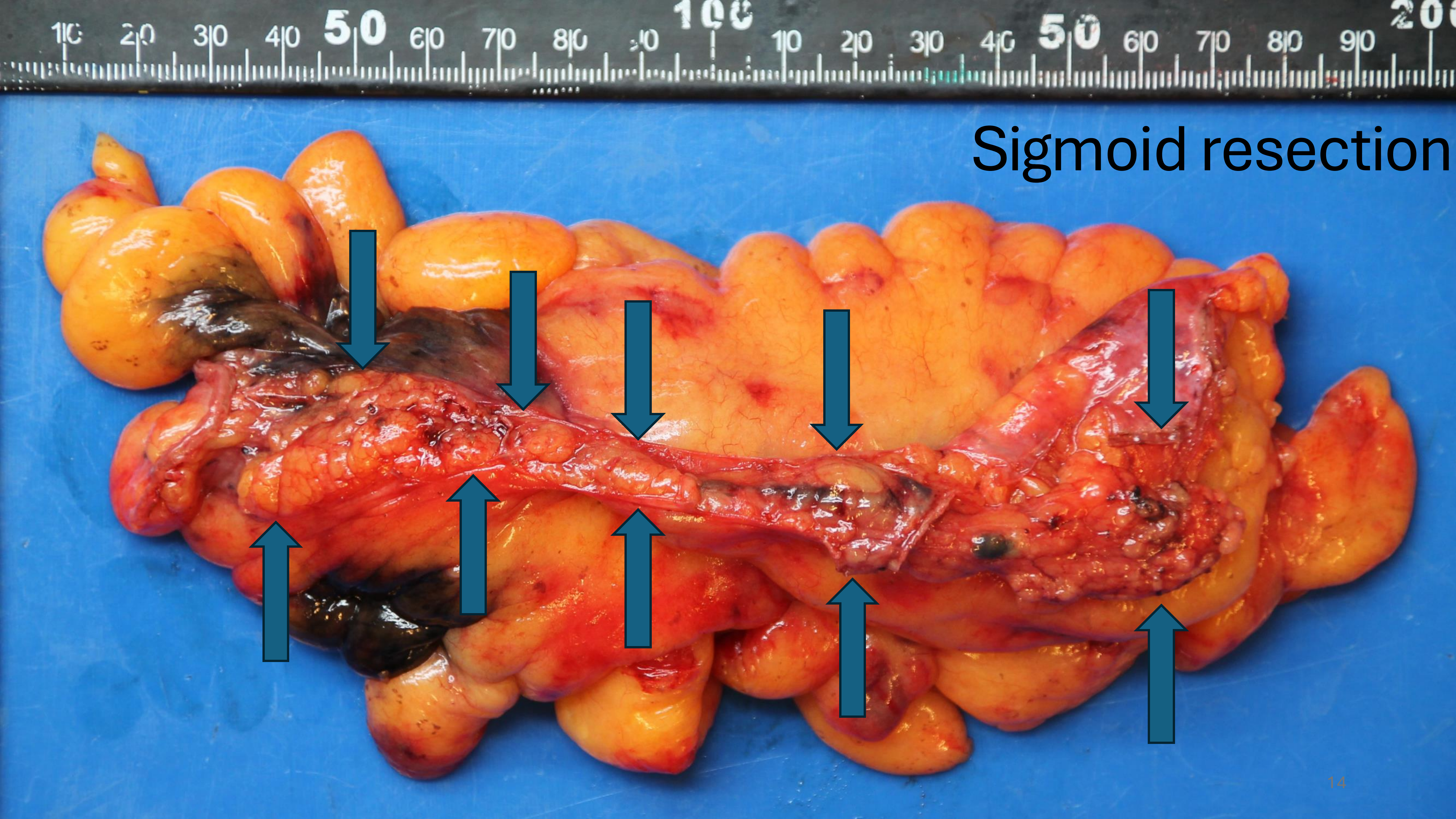

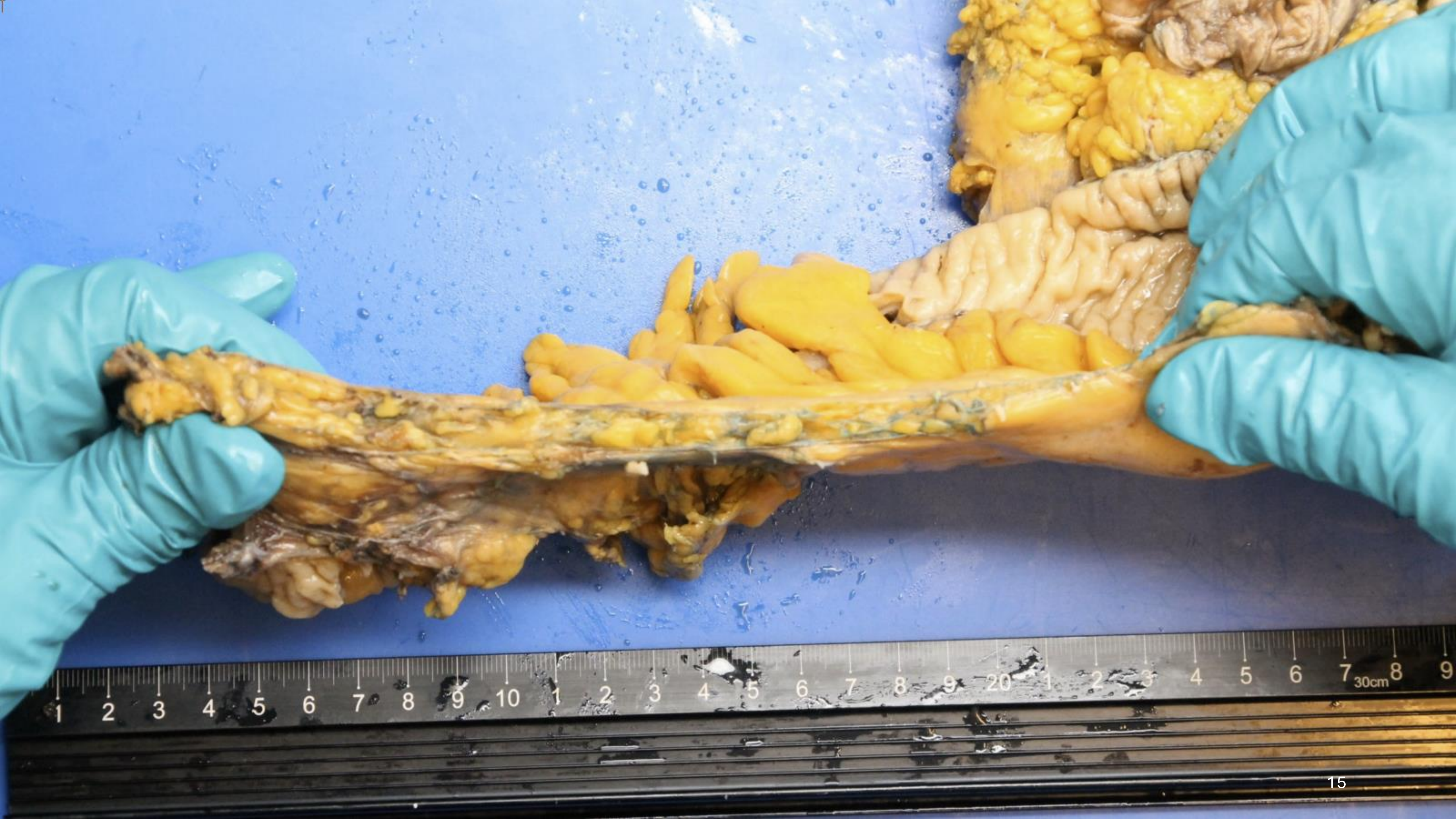

Figures to aid the gross recognition of local peritoneal metastasis in CRC resection specimens

Supplementary data to the paper:

Under-diagnosis of positive resection margins and synchronous peritoneal metastases in locally advanced colon cancer: histopathological reassessment of primary resection in the COLOPEC trial.

ES Zwanenburg MD1,2, DD Wisselink MD1,2, CEL Klaver MD, PhD1,2, JDW van der Bilt MD, PhD1,2,3, JG van den Berg MD, PhD4, LL Kodach MD, PhD4, ID Nagtegaal MD, PhD5, PJ Tanis MD, PhD1,2,6, P Snaebjornsson MD, PhD4,7, on behalf of the COLOPEC collaborators^

Small peritoneal metastasis located in the greater omentum close to the primary tumor colon

A small piece of the greater omentum is normally attached to the first part of the transverse colon

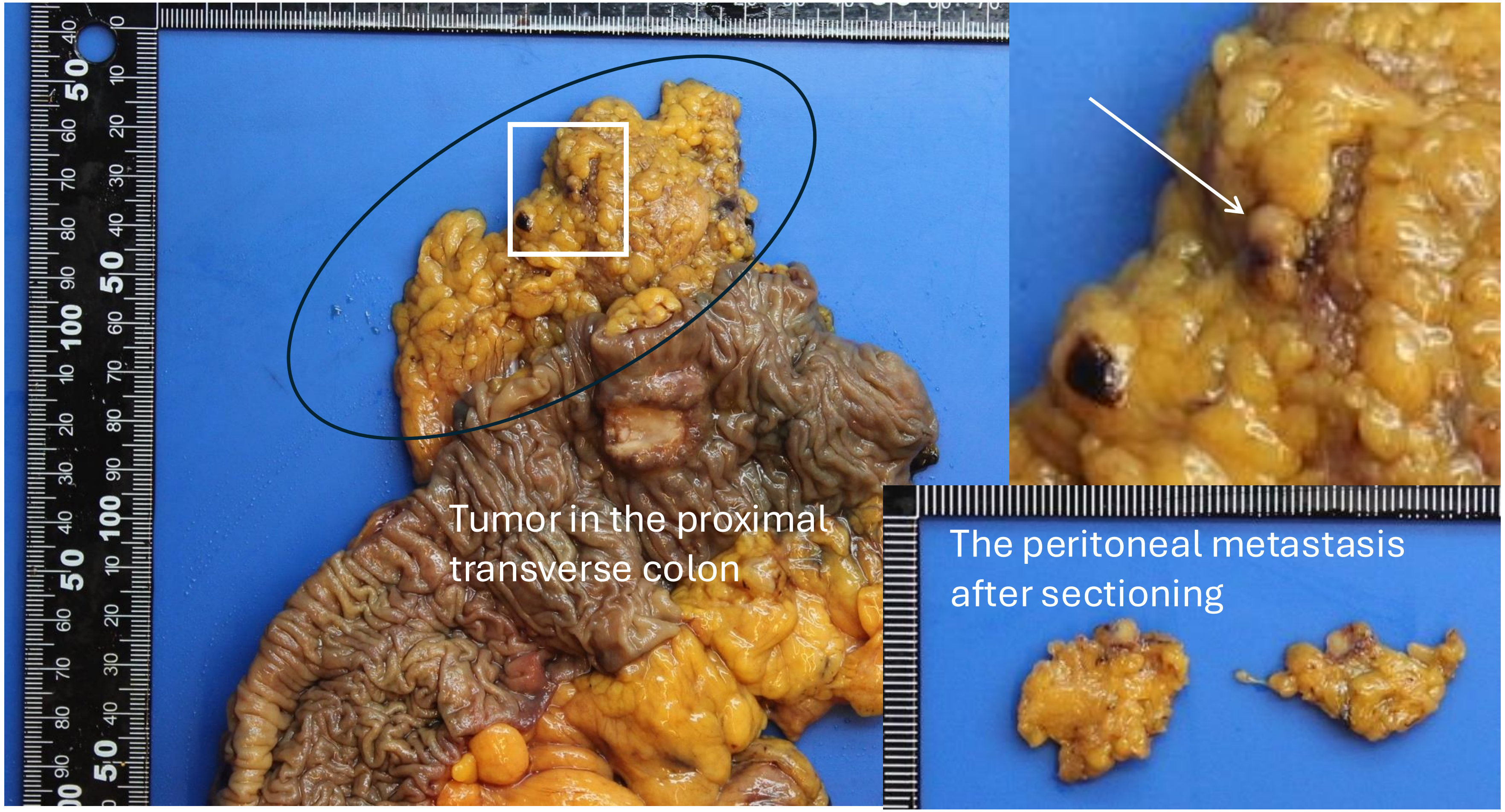

Right hemicolectomy specimen

Exenteration

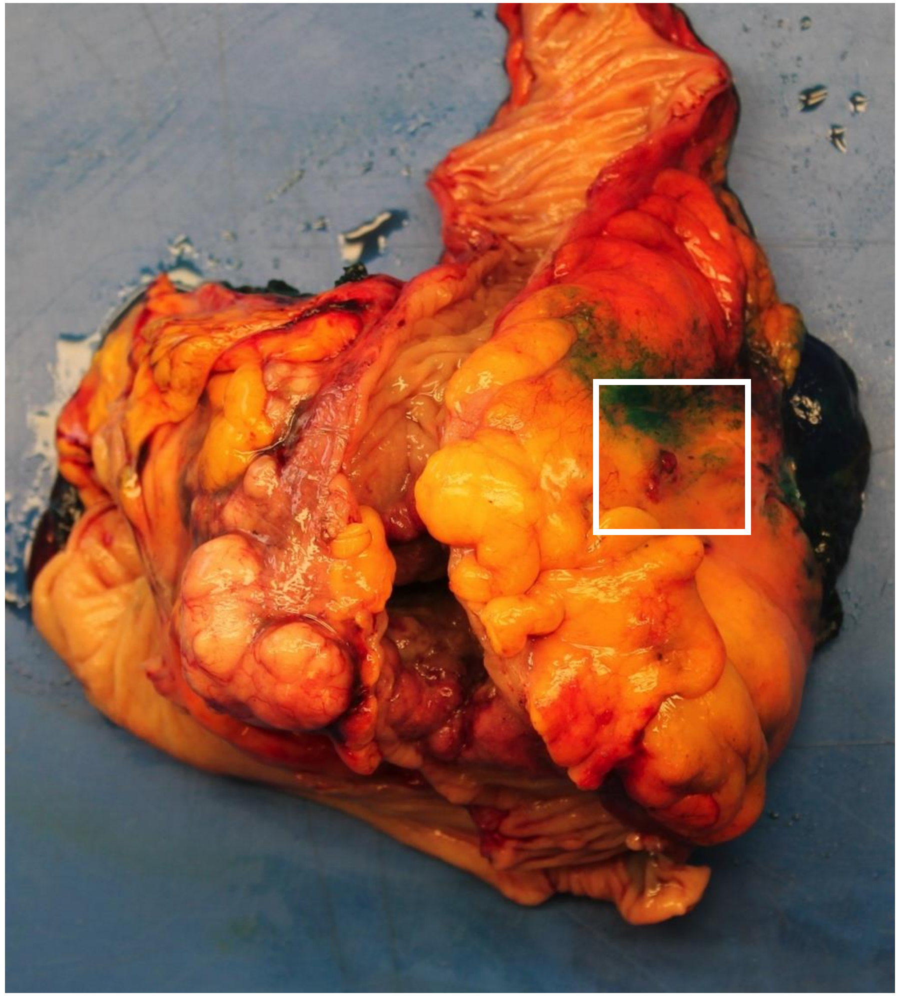

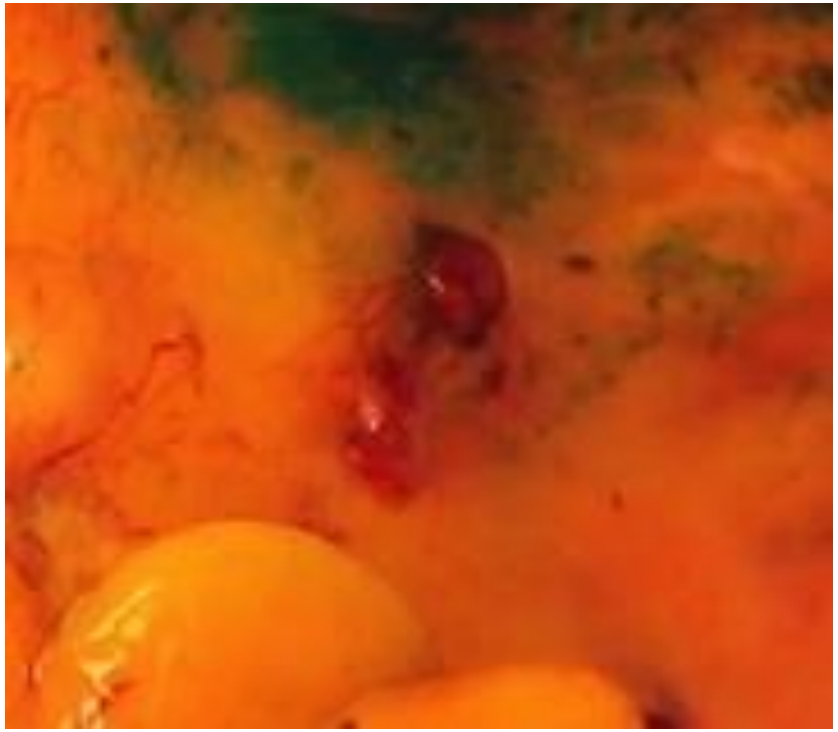

A small peritoneal metastasis within the resection specimen of the primary tumor
This peritoneal metastasis has its center above the peritoneal surface, which is not always the case
