# Supplementary material for: Underdiagnosis of positive resection margins and synchronous peritoneal metastases in locally advanced colon cancer: histopathological reassessment of primary resection in the COLOPEC trial
Source: Virchows Arch. 2025 May 16;487(4):787–97. doi: 10.1007/s00428-025-04065-x (PMC12546376; doi:10.1007/s00428-025-04065-x)
Supplement: Supplementary file 3 — (PDF 682 kb) [file 428_2025_4065_MOESM3_ESM.pdf]

Supplementary data to the paper:

Under-diagnosis of positive resection margins and synchronous peritoneal metastases in locally advanced colon cancer: histopathological reassessment of primary resection in the COLOPEC trial.

ES Zwanenburg MD1,2, DD Wisselink MD1,2, CEL Klaver MD, PhD1,2, JDW van der Bilt MD, PhD1,2,3, JG van den Berg MD, PhD4, LL Kodach MD, PhD4, ID Nagtegaal MD, PhD5, PJ Tanis MD, PhD1,2,6, P Snaebjornsson MD, PhD4,7, on behalf of the COLOPEC collaborators

Suppl. figure 1 Flowchart.

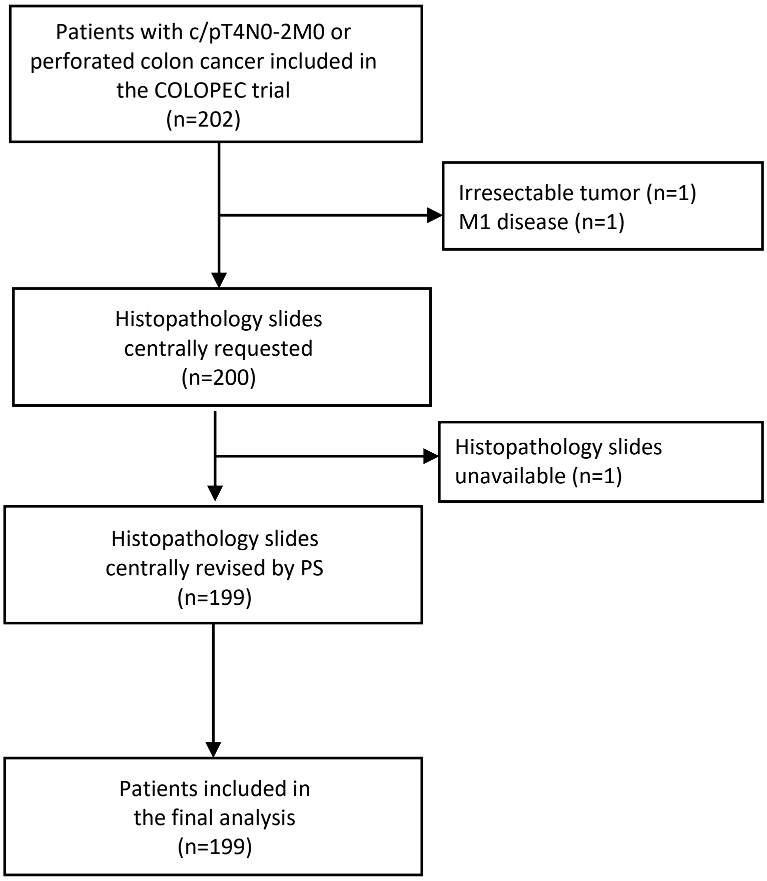

Suppl. figure 2A Kaplan-Meier analysis of 5-year peritoneal metastases, stratified by R0 versus R+.

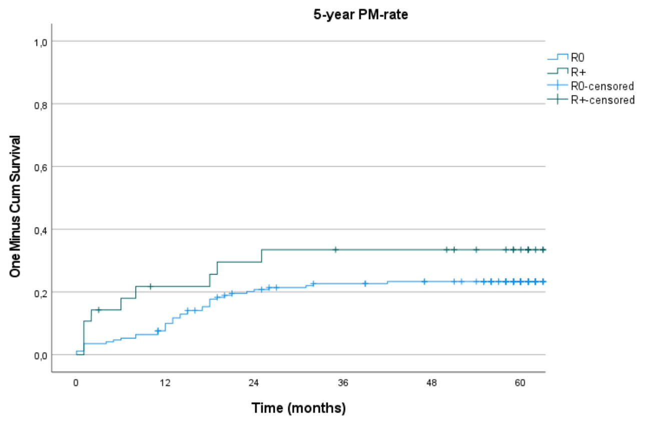

Log Rank, p = 0.179

Number at risk, months	0	12	24	36	48	60
R0	171	155	128	121	116	80
R1	28	20	18	16	16	9

Suppl. figure 2B Kaplan-Meier analysis of 5-year peritoneal metastases, stratified by R+ type.

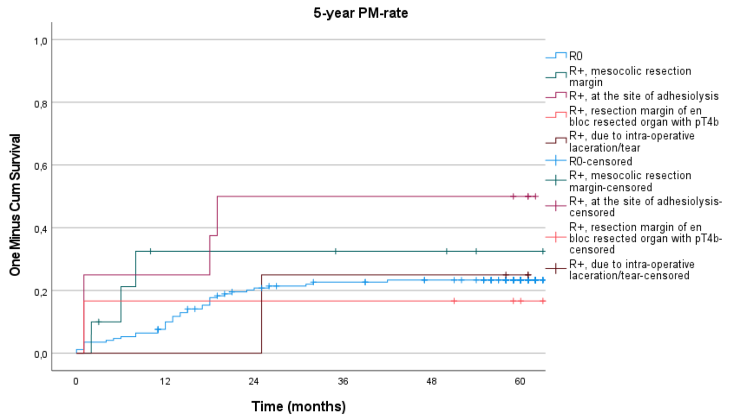

Log Rank, p = 0.321

Number at risk, months	0	12	24	36	48	60
R0	171	155	128	121	117	80
R+ at the mesocolic resection plane	10	5	5	4	4	2
R+ at the site of adhesiolysis	8	6	4	4	4	3
R+ at the resection margin of en-bloc resected structures	6	5	5	5	5	2
R+ due to intra- or postoperative laceration/tear	4	4	4	3	3	2

Suppl. figure 3 Kaplan-Meier analysis of 5-year peritoneal metastases, stratified by presence or absence of SL-PM.

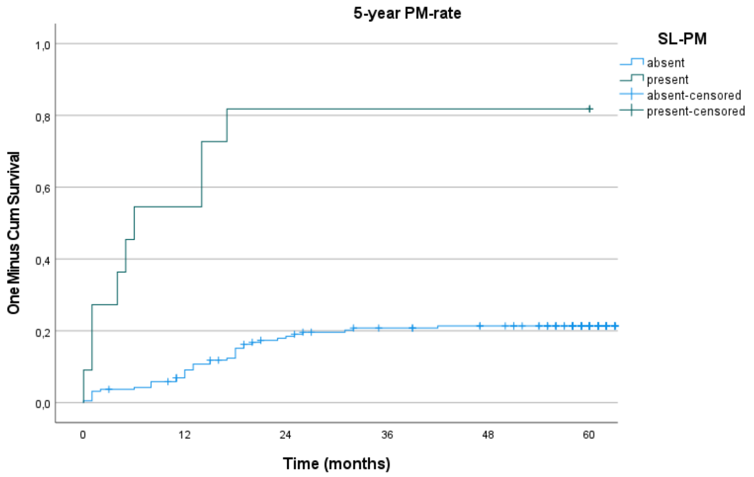

Log Rank, p <0.001

Number at risk, months	0	12	24	36	48	60
SL-PM absent	188	170	144	135	130	88
SL-PM present	11	5	2	2	2	1

Suppl. table 1 Patient, procedure, and revised histopathological tumor characteristics of the total cohort (n=199)

	**Total cohort (n=199)**	
**Patient characteristics**	**n**	**%**
Age >70	25	12.6
Female	95	47.7
ASA-score ≥2	118	59.3
Received adjuvant chemotherapy	174	87.4
Received adjuvant HIPEC	87	43.7
**Procedure characteristics**		
(Extended) right hemicolectomy	76	38.2
(Extended) left hemicolectomy	26	13.1
Sigmoid resection	64	32.2
Subtotal colectomy	4	2.0
Anterior resection	29	14.6
Emergency resection	39	19.6
Completed laparoscopic primary tumor resection	110	55.3
Converted laparoscopic primary tumor resection	20	10.1
Open primary tumor resection	69	34.7

Multivisceral resection	70	35.2
**Tumor and histopathological characteristics**		
Location		
Appendix	5	2.5
Cecum	38	19.1
Ascending colon and hepatic flexure	29	13.1
Transverse colon	12	6.0
Descending colon and splenic flexure	16	6.5
Sigmoid colon	94	47.2
Type and differentation		
Well/moderately differentiated adenocarcinoma	142	71.4
Poorly differentiated adenocarcinoma	24	12.1
Mucinous carcinoma	22	11.1
Signet ring cell carcinoma	11	5.5
pT4a, present*	132	66.3
pT4b, present^	37	18.6
Lymph node status		
pN0	59	29.6
pN1 (1-3 positive nodes)	65	32.7
pN2 (4+ positive nodes)	75	37.7
Tumor deposits, present	56	28.1
Positive resection margin (R+), present	28	14.1
R+, at the site of adhesiolysis	8	28.6
R+, mesocolic resection margin	10	35.7
R+, resection margin of en bloc resected organ with pT4b	6	21.4
R+, due to intra-operative laceration/tear	4	14.3
SL-PM, present	11	5.5

**:tumor cells within <0.1 mm from the free peritoneal surface as defined by Zwanenburg, et al[4]*

^:the presence of pT4b was recorded separate from the presence of pT4a

Suppl. table 2 R+ at the site of adhesiolysis: detailed information from original surgical reports, pathology request forms, initial grossing and microscopic assessment, original pT and R status and microscopic description of R+ status from revision

Excel file

Suppl. table 3 Detailed information regarding cases with synchronous locoregional peritoneal metastases.

Excel file

Suppl. table 4 Cox regression analysis for the association of pathology parameters with 5-year peritoneal metastases as outcome measure: final model after backward selection that included the following parameters as input: pT4a, pN status, R status, SL-PM and adjuvant chemotherapy.

**Variables**	**HR**	**95% CI**
pT4a absent	1	-
pT4a present	2.64	1.20 – 5.80
pN0	1	-
pN1-2	2.93	1.31 – 6.53
R0 resection	1	-
R+ resection	2.38	1.12 – 5.04
SL-PM absent	1	-
SL-PM present	5.98	2.69 – 13.29
Received adjuvant chemotherapy	1	-
Received no ajduvant chemotherapy	6.84	3.04 – 15.41

Suppl. table 5 Characteristics of patients with early peritoneal recurrence at surgical re-exploration for intended prophylactic HIPEC (within 5-8 weeks after the primary tumor resection)

**Histopathology**	**pT4a present***	**R status and type**	**Synchronous locoregional peritoneal metastases present**	**pN**	**Remarks from the surgical report**
Adeno-carcinoma	Yes	R0	No	pN2	No remarks
Adeno-carcinoma	No	R+ at the site of adhesiolysis	No	pN0	Tumor embedded, need for dissection
Adeno-carcinoma	Yes	R0	No	pN2	Tumor adhesive to abdominal wall
Adeno-carcinoma	Yes	R+ at the site of adhesiolysis	No	pN0	Tumor embedded, need for dissection
Signet ring cell carcinoma	Yes	R0	No	pN2	No remarks
Adeno-carcinoma	Yes	R0	No	pN2	Tumor adhesive to mesentery and small bowel
Adeno-carcinoma	No	R+ at the mesocolic resection plane	No	pN2	Tumor embedded, need for dissection
Adeno-carcinoma	Yes	R+ at site of en bloc resection margin pT4b	Yes	pN0	Tumor embedded, need for dissection
Adeno-carcinoma	Yes	R0	Yes	pN2	No remarks

**:tumor cells within <0.1 mm from the free peritoneal surface as defined by Zwanenburg, et al[4]*
